# Chromatin remodeling complexes: architects influencing breast cancer progression

**DOI:** 10.3389/fcell.2025.1690350

**Published:** 2025-11-07

**Authors:** Octavianus Giovani, Grace S. Eckersley, Hayden R. Jones, Sankari Nagarajan

**Affiliations:** Division of Molecular and Cellular Function, School of Biological Sciences, Faculty of Biology, Medicine and Health, The University of Manchester, Manchester, United Kingdom

**Keywords:** epigenetics, chromatin remodeling, breast cancers, aggressiveness, SWI/SNF

## Abstract

As one of the most common types of cancer, breast cancer strongly contributes to the increase in morbidity and mortality worldwide. Alterations in the genetic and epigenetic landscape contribute to the complexity and heterogeneity of the disease, making its understanding and prognosis more challenging. Chromatin remodeling complexes are implicated as essential factors driving the progression and aggressiveness of breast cancer by permitting chromatin dynamics to promote or suppress transcription. Based on their structure and biochemical properties, chromatin remodeling complexes are divided into four subfamilies: SWI/SNF, ISWI, CHD and INO80. Due to their involvement in breast cancer progression, these complexes present potential therapeutic targets, either through direct or indirect approaches. Several promising efforts have been made to develop targeted therapies against chromatin remodeling complexes using specific ATPase inhibitors or proteasome-based degraders to control tumour growth. Further research is needed to elucidate the interplay between the remodeling complexes, their co-regulators, and interacting partners, in order to understand their mechanisms and develop their potential for therapeutic strategies, especially in breast cancer.

## 1 Introduction

Breast cancer is one of the most common cancers in the world, with its morbidity and mortality rates increasing over time (Sung et al., 2021; Siegel et al., 2023). While the burden of other cancers, such as lung cancer and melanoma, is predicted to decrease, breast cancer is expected to create more impact on patients and healthcare systems by 2040 (Arnold et al., 2022). In the UK, there were around 58,000 new cases and 12,000 deaths related to breast cancer in 2022, presenting it as the most common type of cancer (Ferlay et al., 2024). Furthermore, there were a total of 2.3 million new cases and 685,000 deaths associated with breast cancer globally in 2020. These numbers are predicted to climb to over 3 million new cases and 1 million deaths annually by 2040 (Arnold et al., 2022).

## 2 Breast cancer subtypes

Breast cancer is a disease with high heterogeneity, multiple subtypes, and a variety of risk factors, which altogether challenge the diagnosis and treatment of patients, as the different subtypes require different types of therapy. Aggressive breast cancers are associated with metastasis, treatment relapse, and high mortality, hence possessing lower survival rates (Arnold et al., 2022; Afifi and Barrero, 2023). The aggressiveness of breast cancer can be predicted by its subtype, which is based on the expression of the estrogen receptor (ER), progesterone receptor (PR), and the human EGF receptor 2 (HER2). The combination of expression of these three receptors can be used to group breast cancer tumours into four main molecular subtypes: luminal A, luminal B, HER2-enriched, and triple-negative breast cancer (TNBC).

Luminal A cancers show expression of ER and PR (ER+/PR+) but lack HER2 (HER2-) and typically have low numbers of cells exhibiting proliferative markers (e.g., Ki67). They have the most favourable prognosis, as they respond well to ER-targeting therapies. Luminal B cancers are also ER+ and PR+, but they can be either HER2+ or HER2-. They often present with larger, faster-growing tumours with increased Ki67 detection and a slightly worse prognosis than luminal A. HER2 cancers are ER- and PR- but HER2+. They have high rates of proliferation, and patients with these cancers have markedly increased survival rates since the advent of HER2-targeting therapies. TNBC tumours are ER-, PR- and HER2-. They are the most aggressive breast cancers, with poor survival, early metastasis, and limited treatment options (Afifi and Barrero, 2023; Lee et al., 2023).

With the growing knowledge from sequencing technologies, it becomes apparent that in addition to genetic alterations in key proliferative genes (ER, HER2), dysregulation in the epigenetic landscape plays a significant role in driving breast cancer aggressiveness. One type of protein complexes that has been implicated in the epigenetic dysregulation are chromatin remodeling complexes. These complexes are crucial in mediating gene transcription, and alterations in the genes encoding these complex proteins may contribute to oncogenesis and cancer progression several cancers, such as prostate, hepatocellular, pancreatic, colorectal, and oral squamous cell carcinoma (Fang et al., 2011; Pancione et al., 2013; Kumar et al., 2016; Ordonez-Rubiano et al., 2024). This review aims to elaborate in-depth on the functions of the chromatin remodeling complexes, explore their roles in driving progression and aggressiveness in breast cancer, and discuss how inhibitors of chromatin-modifying proteins have shown promise as an alternative treatment for breast cancer (Table 1).

**TABLE 1:** Table showing the key results and function of chromatin remodeling complex subunits in breast cancer.

Complex	Subunit	Key results	Proposed functional classification	References
SWI/SNF	BRG1/BRM​	Functional studies: Promotes cell proliferation and fatty acid production Enhances colony formation, migration, invasion and cell viability Regulates cell cycle Non-functional studies: Highly expressed in primary breast cancer	Oncogenic	Wu et al, 2015 Bai et al. 2013 Wu, 2012 Li et al. 2024a Sobczak et al. 2020 DiRenzo et al. 2000 Wu et al. 2016a
	ARID1A	Functional studies: Suppresses migration and colony formation Influences cell proliferation Associated with drug sensitivity Non-functional studies: 78% of TNBCs exhibit low ARID1A expression Low ARID1A mRNA expression is associated with advanced tumors, increased p53 expression, and high Ki-67 expression	Tumor suppressive	Nagarajan et al. 2020 Guo et al. 2018 Xu et al. 2020 Zhang et al. 2012
	ARID1B	Functional studies: Supports cell proliferation Non-functional studies: ARID1B is highly expressed in TNBC	Oncogenic	Cui et al. 2019 Shao et al. 2015 Stephens et al. 2012
	PBRM1	Functional studies: Inhbits cell proliferation Non-functional studies: Low expression of PBRM1 in breast cancer tissues is associated with poor prognosis	Tumor suppressive	Xia et al. 2008 Mo et al. 2015
	ARID2	Non-functional studies: Reduced ARID2 expression is frequently found in non-luminal breast cancer subtypes Reduced ARID2 is a predictor of poor survival in ER-positive breast cancer patients	Tumor suppressive	Zhang et al. 2021
ISWI	SMARCA5	Functional studies: Supports cell proliferation and invasion, impacting cell cycle and senescence	Oncogenic	Jin et al. 2015 Li et al. 2019
	SMARCA1	Functional studies: Enables cell proliferation and cell survival	Oncogenic	Ye at al. 2009
	BAZ1A	Functional studies: Associated with cellular senescence Non-functional studies: Associated with poor overall survival and relapse-free survival Amplification is associated with short metastasis free survival	Oncogenic	Pérez-Pena et al. 2019 Li et al. 2019
	BPTF	Functional studies: Enhances cell proliferation and cell survival Non-functional studies: Amplification is associated with advanced tumor grade in ER+ and TNBC	Oncogenic	Bezrookove et al. 2022
	CECR2	Functional studies: Supports migration, invasion and metastasis	Oncogenic	Zhang M. et al. 2022
CHD	CHD1	Functional studies: Facilitates cell proliferation	Oncogenic	Tan et al. 2014
	CHD3	Non-functional studies: CHD3 showed heterozygous loss in approximately 60% of breast cancer.	Tumor suppressive	Chu et al. 2017
	CHD4​	Functional studies: Enables proliferation and cell survival Supports migration, colony formation and invasion Influences EMT	Oncogenic	Hou et al. 2017 Luo et al. 2018 Wang et al. 2020 D'Alesio et al. 2016 D'Alesio et al. 2019
	CHD5	Non-functional study: CHD5 loss-of-function has been reported in breast cancer pathogenesis	Tumor suppressive	Wu et al. 2012
	CHD7	Non-functional studies: Amplification of CHD7 represents around 11% breast cancer patients Amplifications are more prevalent in aggressive breast cancer subtypes, correlating with high tumor grade and poor prognosis.	Oncogenic	Chu et al. 2017
	CHD9	Non-functional studies: CHD9 showed heterozygous loss in approximately 55% of breast cancer.	Tumor suppressive	Chu et al. 2017
INO80	INO80	Non functional studies: INO80 expression is significantly downregulated in basal-type breast cancer Reduced expression of INO80 is associated with reduced overall survival rate, distant metastasis-free survival, and recurrence-free survival in breast cancer patients.	Tumor suppressive	Thang et al. 2023
	SRCAP	Functional studies: Moderates proliferation and invasion	Oncogenic	Cao et al. 2022

## 3 Chromatin remodeling complexes

**FIGURE 1:**
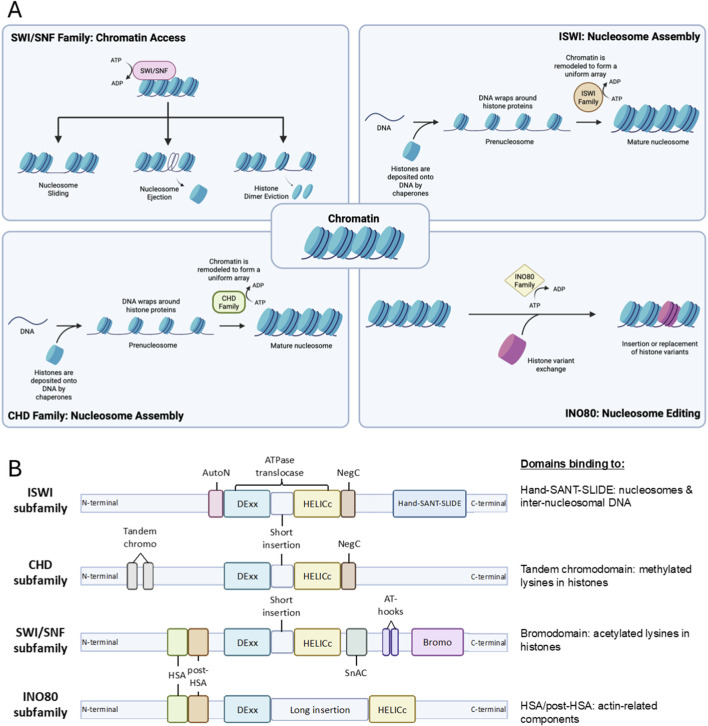
Schematic depiction of chromatin remodeling complexes. **(A)** General functions of chromatin remodeling complexes, which include nucleosome maturation assembly and spacing to form mature nucleosome by ISWI and CHD complexes, promoting chromatin acssibility by enabling chromatin repositioning through nucleosome sliding, nucleosome ejection and histone dimer eviction by SWI/SNF complex, and nucleosome editing *via* insertion or canonical and variant histone performed by INO80 complex. **(B)** Structure of each subfamily proteins with their domains. Each possesses split ATPase domains composed of Dexx and HELICc segments, with different domains unique to each subfamily. HSS and Bromo domains near the C-terminals of ISWI and SWI/SNF subfamilies bind to nucleosomes and histones of interest. Tandem chromo and HSA/post-HSA domain adjacent to N-terminals of CHD and INO80 subfamilies bind to methylated lysines in histone and actin-related components, respectively.

Gene expression is vital for cells to function and survive. However, the intricacy of chromatin architecture and the position of nucleosomes along the DNA strands limit the accessibility of DNA for transcription factor binding and therefore control gene expression. The chromatin remodeling complexes are comprised of enzymes and proteins that regulate chromatin accessibility by opening or closing the chromatin to execute their functions on replication, gene expression, repair, and recombination (Kadoch and Crabtree, 2015). These complexes function by establishing the proper density and spacing of nucleosomes, which leads to the packaging and unpackaging of chromatin during the aforementioned processes (Clapier and Cairns, 2009; Clapier et al., 2017). Hence, based on their function, remodelers can be classified into three groups: nucleosome assembly and organization, chromatin access, and nucleosome editing (Figure 1A) (Flaus et al., 2006; Clapier et al., 2017).

Most remodeling complexes use ATP hydrolysis to alter the histone-DNA contacts. They are more extensively studied and share several properties that enable the engagement, selection, and remodeling of the nucleosome. These properties include the affinity for nucleosomes compared to the DNA elements, domains that recognize covalent histone modifications, catalytic subunits with ATPase domains to break histone-DNA contacts, domains that regulate the ATPase domain, and domains for interaction with other chromatin or transcription factors for selective action on particular nucleosomes at specific sites (Clapier and Cairns, 2009; Clapier et al., 2017).

**FIGURE 2:**
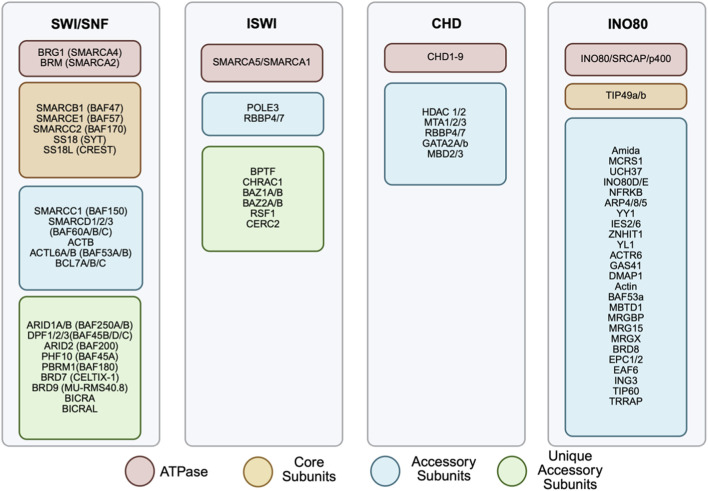
Subunits composition of chromatin remodeling complexes. The four chromatin remodeling complexes: SWI/SNF, ISWI, CHD and INO80, are represented with their respective subunits listed. Each family is made up of a selection of subunits including ATPase (red), core subunits (yellow), accessory subunits (blue) and unique accessory units (green). Different combinations of each subunit can result in differing complex actions. Several of these subunits have been implicated in cancer.

This review will focus on the members of ATP-dependent chromatin remodeling complexes comprising SWItch/Sucrose Non-Fermentable (SWI/SNF), Imitation SWItch (ISWI), Chromodomain Helicase DNA-binding protein (CHD), and INOsitol-requiring mutant 80 (INO80) remodeler families (Figure 2). Despite their similar properties, they possess differences in ATPase structure and complex constituencies (Figure 1B). For example, the ATPase domains of all remodeler families are divided into DExx and HELICc segments, but the INO80 family has a unique longer insertion domain between the two segments. Other distinguishing features are the differences in the combinations of flanking domains within the remodeler families, such as bromodomain for the SWI/SNF family, Helicase and SWI-3 adaptor 2 Nuclear receptor co-repressor Transcription factor IIIB (SANT)-associated (HSA) domain for the SWI/SNF and INO80 families, HAND-SANT-SLIDE (HSS) module for the ISWI family and tandem chromodomains for the CHD family. These domains are evolutionarily conserved in protein composition and function (Clapier and Cairns, 2009).

### 3.1 SWI/SNF subfamily

SWI/SNF remodelers can slide nucleosomes along DNA, evict nucleosome components and eject whole nucleosomes, rendering the chromatin more accessible to proteins and RNA. This exposes binding sites for transcription factors, coactivators or repressors as well as DNA repair and recombination factors (Boeger et al., 2004; Clapier and Cairns, 2009; Clapier et al., 2017).

#### 3.1.1 Structure and composition

**FIGURE 3:**
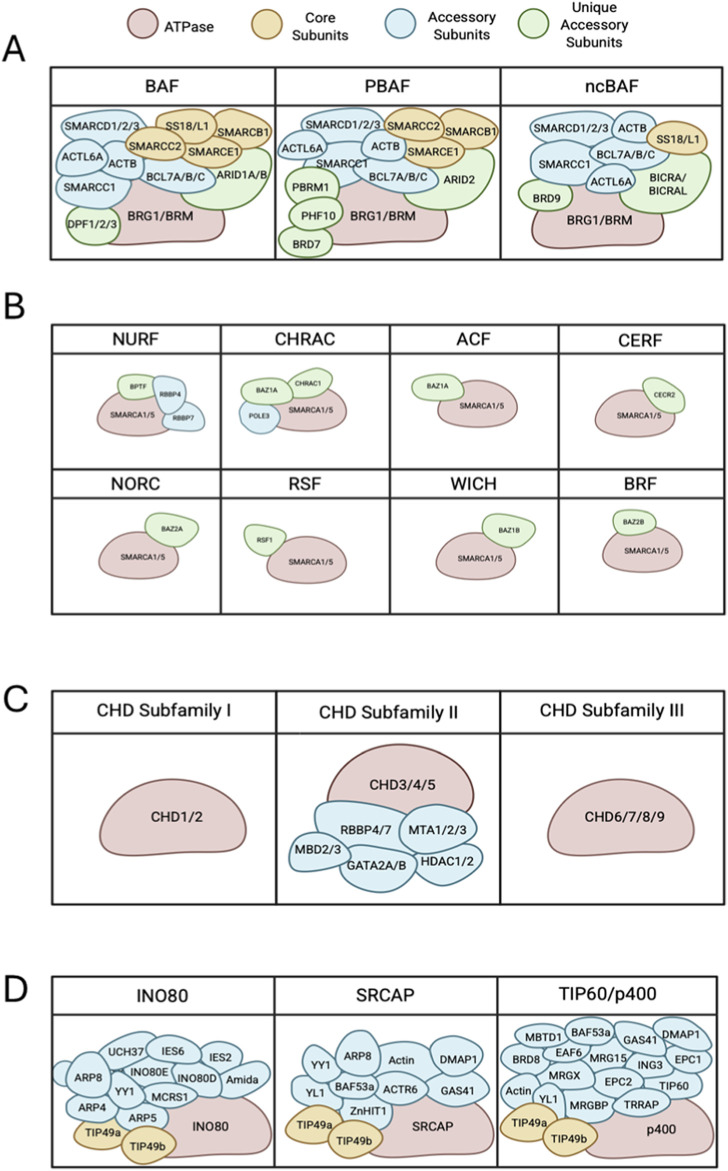
Subunit and domain arrangement of chromatin remodeling complex subfamilies. **(A)** All SWI/SNF complexes share BRG1 or BRM as ATPase subunits (red) that complex with various core subunits (yellow), accessory subunits (blue) and several unique accessory subunits (green) forming BAF, PBAF and ncBAF. **(B)** ISWI subfamilies are comprised of SMARCA5 or SMARCA1 as the core ATPase subunits (red), and accessory proteins (blue) and unique accessory subunits (green), making up to 16 complexes. **(C)** Chromatin remodelers in the CHD family can be divided into 3 subfamilies. Subfamily 1 and 3 consist of the core CHD ATPase. CHD subfamily 2 reflects a larger complex of multiple accessory subunits (blue). **(D)** Remodelers of INO80 subfamilies have several proteins that bind to scaffold domains including an ATPase (red), core subunits (yellow) and accessory subunits (blue).

The SWI/SNF family is composed of a central catalytic subunit with 8–14 associated subunits. The catalytic subunit possesses an ATPase-translocase domain formed by two RecA-like lobes that flank a small, conserved insertion, surrounded by an N-terminal HSA domain that binds to actin or actin-related proteins (ARP), post-HSA domain, AT-hooks, and C-terminal bromodomain (Figure 1B) (Mohrmann and Verrijzer, 2005; Kasten et al., 2011; Schubert et al., 2013; Clapier et al., 2017). There are three SWI/SNF complexes in humans: BRG1-associated Factors (BAF, SWI/SNF-A), Polybromo-associated BAF (PBAF, SWI/SNF-B), and the recently defined non-canonical BAF (ncBAF) (Xue et al., 2000; Mohrmann and Verrijzer, 2005; Mani et al., 2017). Each functional complex can only possess one catalytic subunit, either Brahma [BRM/SWI/SNF related, matrix-associated, actin-dependent regulator of chromatin, subfamily A, member 2 (SMARCA2)] or Brahma-Related Gene (BRG1/SMARCA4). The rest of the complex is formed by core subunits, which are essential for remodeling activity, and accessory subunits that target or regulate the complex activity (Figures 2, 3A).

The core subunits include SMARCB1 (SNF5), SMARCE1, SMARCC2, Synovial Sarcoma Translocation Gene on Chromosome 18 (SS18), and SS18-Like 1 (SS18L1). Some accessory subunits are common in major subfamilies, such as SMARCC1, SMARCD1/2/3, β-actin, Actin Like 6A/B (ACTL6A/B), and B-Cell Lymphoma 7 Protein Family Member A/B/C (BCL7A/B/C). The remaining accessory subunits act as signature unique subunits, which define the three SWI/SNF complexes. These specific subunits include AT-Rich Interactive Domain-Containing Protein 1A/1B (ARID1A/ARID1B) and Doubled PHD Fingers Family 1/2/3 (DPF1/2/3) for BAF; ARID2, PHD Finger Protein 10 (PHF10), Polybromo 1 (PBRM1), and Bromodomain Containing 7 (BRD7) for PBAF; and BRD9, BRD4 Interacting Chromatin Remodeling Complex Associated Protein (BICRA), and BICRA-Like (BICRAL) for ncBAF (Mohrmann and Verrijzer, 2005; Mani et al., 2017). The core subunits of SWI/SNF can be combined with different specific subunits, which results in tissue- and developmental-specific remodeler subtypes (Ho and Crabtree, 2010; Clapier et al., 2017). BAF subunits bind strongly on enhancer regions, while PBAF and ncBAF subunits are mostly enriched on promoters (Nakayama et al., 2017; Gatchalian et al., 2018; Schick et al., 2019; Wang et al., 2019; Mittal and Roberts, 2020).

#### 3.1.2 Role in breast cancer

Among the four remodeling complexes, the SWI/SNF complex is the most studied, with the strongest link in various cancers (Varela et al., 2011; Wilson and Roberts, 2011). In general, the SWI/SNF complex is thought to be a tumor suppressor (Jones et al., 2022). Around 20% of all cancers possess mutations in the genes encoding SWI/SNF subunits (Kadoch et al., 2013). The mammalian SWI/SNF complex comprises subunits that require each other for redundant/non-redundant genomic functions. Hence, any changes in the concentration of the subunits may lead to aberrant gene and transcription activity, which can eventually expedite cancer progression (Wu et al., 2017; Cui et al., 2019; Jones et al., 2022).

Contrary to the tumor suppressive role of the SWI/SNF complex, the ATPase subunits of the SWI/SNF complex, BRM and BRG1, are highly expressed in primary breast cancer compared to normal breast tissue and are vital for cell proliferation (Table 1) (Bai et al., 2013; Wu et al., 2015). BRG1 is known to play various essential roles, but it can act either as a tumor suppressor or a tumor promoter in different types of breast cancer (Wu, 2012; Wu et al., 2015; Li K. et al., 2024). As a tumor suppressor, BRG1 can interact with another tumor suppressor, BRCA1, to stimulate the transcription of *TP53* or regulate TP53 directly (Bai et al., 2013; Sobczak et al., 2019; Sobczak et al., 2020). On the other hand, BRG1 can also promote cell proliferation by associating with E2F transcription factors on gene promoter sites (Li K. et al., 2024). In addition, BRG1 is required as a coactivator in ER signaling. BRG1 is recruited in an estrogen-dependent manner to enhancer DNA regions containing estrogen-responsive elements (EREs), which show ERα binding and possess active marks such as histone acetylation (DiRenzo et al., 2000). The chromatin remodeling mediated by BRG1 and the activity of histone acetyltransferases such as CBP, p300, and p300/CBP-associated factor (PCAF) on these enhancers lead to nuclear hormone receptor-dependent transcriptional activation (Sobczak et al., 2020). Furthermore, BRG1 is vital in supporting proliferation by promoting fatty acid synthesis in TNBCs (Wu et al., 2016a; Li K. et al., 2024). On the other hand, *BRM* loss promotes proliferation and drives the transformation of normal epithelial cells (Cohet et al., 2010). A study by Yang et al. further confirmed that the expression level of *BRM* is inversely correlated with breast cancer malignancy. This occurs due to the epigenetic activation of Claudins, a family of tight junction proteins, which further suppresses the migration and invasion of breast cancer cells (Yang et al., 2019). However, various knockout and knockdown studies on *BRG1* and *BRM* further confirmed its role in maintaining cellular proliferation, in which the absence or reduced expression of *BRG1* or *BRM* significantly decreases the proliferation rate of breast cancer cells in both *in vivo* and *in vitro* models (Bai et al., 2013; Wu et al., 2015). The effect of BRG1 or BRM perturbations on cell proliferation might be mediated through independent mechanisms, as the knockdown of each gene reduced cell viability independently, while combined knockdown resulted in an additive effect on cell proliferation.

**FIGURE 4:**
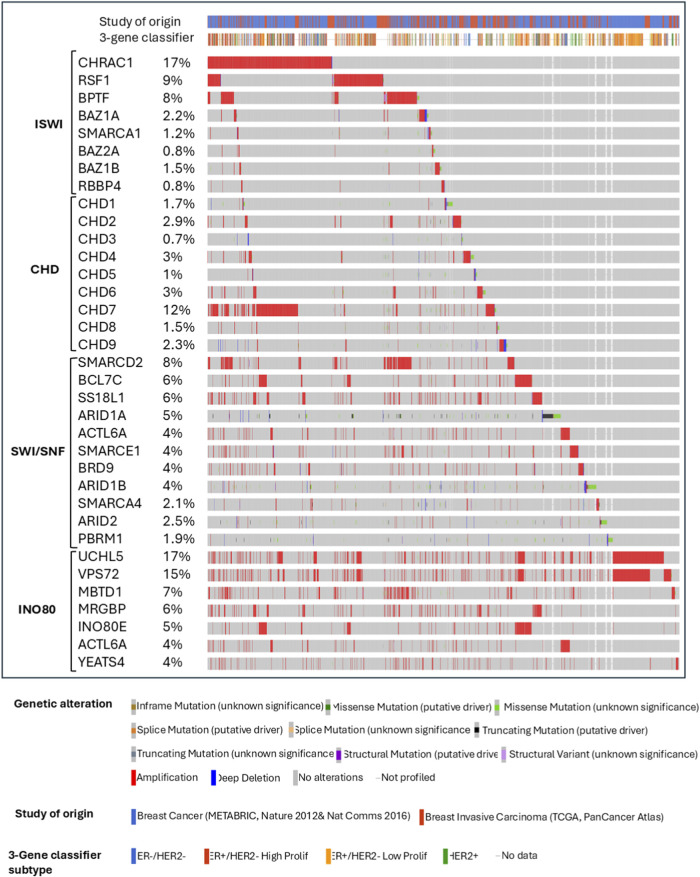
Summary plot of breast cancer study cohorts showing the most altered ISWI, CHD, SWI/SNF, and INO80 complex genes from the chromatin remodeling complexes in patients from METABRIC and TCGA invasive breast cancer studies from cBioPortal.

SMARCD2, BCL7C and SS18L1 are amplified in 6%–8% of breast cancer patients from the TCGA and METABRIC breast cancer patient cohorts (Figure 4) (Curtis et al., 2012; Weinstein et al., 2013). Immunohistochemistry (IHC) analysis by Tropee et al. in breast cancer patient samples further signifies the possibility of SMARCD3’s role in tumour suppression through its association with cell cycle regulators (Tropée et al., 2021). While SMARCD2 is known to play a crucial role in regulating chromatin accessibility and is amplified in many patients (Figure 4), evidence regarding its role in breast cancer remains scarce and needs further exploration.

Besides the core subunits, significant aberration of the SWI/SNF complex activity may be caused by mutations in auxiliary subunits. *ARID1A* and *ARID1B* in the BAF subfamily are frequently mutated in breast cancer (Li K. et al., 2024). The majority of *ARID1A* mutations are heterozygous deletions, but they show biallelic inactivation of the gene due to epigenetic mechanisms, potentially leading to complete protein loss in IHC analyses in patients (Priestley et al., 2019). ARID1A and ARID1B may influence breast cancer by promoting various cellular functions related to tumor suppression, hence controlling aggressiveness and therapeutic response (Li K. et al., 2024). Moreover, approximately 58% of the *ARID1A* and *ARID1B* mutations are known to occur in their intrinsically disordered regions (IDRs) (Patil et al., 2023). These IDRs are heterogeneous conformational ensembles that engage in non-specific interaction compared to other folded domains (Konrat, 2014; Piovesan et al., 2021). Functionally, IDRs are considered to be responsible for influencing the dynamics of chromatin-bound proteins, and creating transcription hubs or phase-separated dense condensates, leading to transcriptional regulation (Boija et al., 2018; Sabari et al., 2018; Wei et al., 2020). Hence, overcoming the challenges in targeting IDRs may present a promising therapeutic strategy for treating breast cancers.

ARID1A maintains various vital cellular functions, including cell cycle control, preserving genomic integrity during double strand break repair processes, preventing telomere lengthening (Li and Lee, 2023). ARID1A is also known to participate directly in DNA repair by promoting DNA end resection (Davó-Martínez et al., 2023). ARID1A is known to perform its role by mobilizing nucleosomes to create nucleosome-depleted regions, activating the binding of transcription factors, and thus enabling gene transcription. On the other hand, ARID1A associates with nuclear hormone receptors on enhancers to control the expression of estrogen target genes and cell cycle regulators (Nagarajan et al., 2020). Other studies also demonstrate that ARID1A is critical in mediating chromatin accessibility on promoters and enhancers and thus regulates their activity (Alver et al., 2017; Mathur et al., 2017). Therefore, loss-of-function mutation in *ARID1A* may compromise the expression of many genes involved in tumor suppression, differentiation, Epithelial to Mesenchymal Transition (EMT), and lineage specificity, which subsequently leads to cancer progression. This is supported by an *in vitro* migration assay study, which found that ARID1A effectively inhibits cell migration in multiple breast cancer cell lines (Guo et al., 2018). In addition, *ARID1A* loss-of-function is also correlated with the presence of activating mutations in the gene of Phosphatidylinositol-4,5-Bisphosphate 3-Kinase Catalytic Subunit Alpha *(PIK3CA*), loss of Phosphatase and Tensin Homolog *(PTEN*) expression, and loss of p53 function (Yamamoto et al., 2012; Zang et al., 2012; Bosse et al., 2013; Allo et al., 2014). A clinicopathological analysis also revealed that 78% of TNBCs exhibit low *ARID1A* expression, and breast cancer with low *ARID1A* mRNA expression is associated with advanced tumors, increased p53 expression, and high Ki-67 expression (Zhang et al., 2012).

Interestingly, in metastatic, drug-relapsed or endocrine-resistant ER-positive breast cancer patients and primary lobular breast cancer patients, who are generally resistant to ER-targeted therapies, *ARID1A* inactivating mutations are more frequently observed, suggesting that *ARID1A* loss is sufficient to mediate therapy resistance (Desmedt et al., 2016; Yates et al., 2017; Razavi et al., 2018; Nagarajan et al., 2020; Xu et al., 2020; Schwartz et al., 2021). Consistently, ARID1A is known to control therapeutic response in breast cancer cell lines by influencing the innate proliferative potential, as shown by Nagarajan et al. (2020). In this study, ER+ breast cancer cells with ARID1A loss showed increased tumor growth in both untreated and 4-hydroxytamoxifen (a selective estrogen receptor modulator)-treated cells compared to *ARID1A* wild-type. Using Chromatin Immunoprecipitation assay with sequencing (ChIP-seq) analyses, it was observed that ARID1A binding decreases with estrogen and increases after 4-hydroxytamoxifen treatment, and its loss led to upregulation in the expression of tamoxifen-response genes, suggesting ARID1A as a corepressor and tumor suppressor. The effect on endocrine resistance can also be explained by the increased chromatin accessibility and decreased binding of histone deacetylase 1 (HDAC1) following the loss of ARID1A, which supposedly promotes the transcriptional process. The resulting hyperacetylation on histone H4K5, 8 and 12 residues (but not H3K27) due to HDAC1 loss leads to increased binding of a Bromodomain and Extra-Terminal domain (BET) containing protein, BRD4, which influences subsequent BRD4-dependent transcription (Nagarajan et al., 2020). Interestingly, a lineage-specific pioneer factor Forkhead Box A1 (FOXA1) promotes ARID1A binding on a subset of enhancers, while estrogen stimulation and its subsequent ER activation regulate ARID1A binding (Nagarajan et al., 2020). The findings from this work were concordant with a similar study by Xu et al. on *ARID1A* knockout in breast cancer cells deprived of estrogen, which showed increased proliferation compared to controls, suggesting a role in estrogen-independent proliferation (Xu et al., 2020). Moreover, ARID1A depletion *in vitro* leads to resistance towards Fulvestrant (a selective estrogen receptor degrader). This process is modulated through the reprogramming of chromatin accessibility and chromatin landscape of the breast cancer cells. In contrast to Nagarajan et al., Xu et al. identified that the loss of ARID1A induces a reduction in H3K27ac levels, which act as a strong marker for active enhancers and promoters, in several sites that have lost chromatin accessibility. Moreover, *ARID1A* loss disrupts the occupancy of several transcription factors, including master regulators of ER-dependent transcription and the key determinants of luminal phenotypes, such as ER, FOXA1, and GATA3, while at the same time increasing the binding of TEA Domain Transcription Factor 4 (TEAD4), which is more enriched in the basal phenotype. *TEAD4* silencing is associated with the sensitivity of *ARID1A* knockout cells to fulvestrant. Furthermore, gene set enrichment analysis (GSEA) by Xu et al. upon *ARID1A* knockout reported a significant activation of basal-like transcription, as reflected by the increased activity of stemness-related gene expression such as keratins *KRT6*, *KRT15, KRT5, CD44*, and Tumor Protein P63 (*TP63*). Altogether, the findings from Xu et al., indicated a cellular plasticity of luminal to basal transition upon *ARID1A* loss, making the cells resistant to endocrine therapies.

ARID1B is the homologous counterpart of ARID1A with similar function. Hence, *ARID1A-*mutated tumours may depend on ARID1B for proliferation, suggesting a synthetic lethality combination between ARID1A and ARID1B and thus providing an opportunity for personalised targeting of cancer growth (Nagl et al., 2007; Helming et al., 2014). Given its function in regulating the cell cycle, the above studies showed that ARID1B is an essential subunit of the SWI/SNF complex, with a broad role in promoting proliferation and cell cycle progression. Various evidence illustrates that inactivating mutations in *ARID1B* enable the clonal selective advantage in cancer cells, especially in breast cancer with drug relapse (Stephens et al., 2012). ARID1B also acts as a prognostic biomarker whose expression is associated with disease-free survival in breast cancer patients (Cui et al., 2019; Li K. et al., 2024). On the other hand, *ARID1B* is highly expressed in TNBC compared to other molecular types of breast cancer. Its high expression is closely related to the clinicopathological profile of the patients, such as age, tumor size, histological grade, and nuclear polymorphism, and serves as an independent predictive marker (Shao et al., 2015).

Similar to the BAF subfamily, the mutations in auxiliary subunits in the PBAF subfamily, such as *PBRM1* and *ARID2*, are frequently observed in breast cancers (Li K. et al., 2024). *PBRM1* is a tumor suppressor gene that binds to the p21 promoter and upregulates its baseline and signal-dependent transcription, subsequently inhibiting tumor development in breast cancer cells (Xia et al., 2008). PBRM1 interaction with p21 protein is also implicated in various functions, including DNA damage response, cell cycle regulation, cellular differentiation, and maintenance of genomic stability (Hopson and Thompson, 2017; Hagiwara et al., 2021; Yamashita et al., 2023). However, PBRM1 is known to promote resistance to T-cell-dependent killing in preclinical cancer models, hence the possibility of affecting the anti-tumor immune response (Miao et al., 2018; Pan et al., 2018). Several studies identified PBRM1 as a prognostic indicator in breast cancer patients. PBRM1 expression is strongly correlated with clinical stage and lymph node status and serves as a significant indicator for overall survival and recurrence-free survival in breast cancer patients. Low expression of *PBRM1* in breast cancer tissues is associated with an unfavorable outcome (Mo et al., 2015).

Despite sharing the same AT-rich interaction domain and some structural similarities, ARID2 is not a homolog of BAF subunits ARID1A and ARID1B (Li K. et al., 2024). A decrease in *ARID2* expression is frequently found in non-luminal breast cancer subtypes but ARID2 is a predictor of poor survival in ER-positive breast cancer patients (Zhang et al., 2021). Furthermore, a genomic analysis in matched primary and metastatic breast cancer tissues by Yates et al. found that *ARID1A, ARID1B*, and *ARID2* are often inactivated in recurrent breast cancer. This finding is of significant interest since these genes are commonly wild-type in primary breast cancer and imply selective treatment-induced clonal evolution of aggressive cells with these mutations (Yates et al., 2017). Further investigation on the role of ARID2 in breast cancer would be of great interest.

While SWI/SNF is overall considered a tumor suppressor, some members of the subfamily possess dual roles as tumor suppressors and oncogenes in certain cancers (Table 1) (Sen et al., 2019; Fontana et al., 2023), but the mechanism underlying this context-specific function is unknown.

### 3.2 ISWI subfamily

ISWI subfamily is closely related to the SWI/SNF and is mainly responsible for nucleosome assembly and organization following DNA replication and transcription at locations where the nucleosomes are ejected (Clapier et al., 2017). Upon chromatin disassembly (e.g., in the replication process), histone H3-H4 tetramers and H2A-H2B dimers were brought to adjacent DNA by histone chaperones. ISWI remodelers then assist in forming prenucleosomes with DNA-histone combinations and their maturation into nucleosomes with DNA-histone octamers (Torigoe et al., 2011; Gurard-Levin et al., 2014; Fei et al., 2015; Clapier et al., 2017). Subsequently, by placing them at a fixed and appropriate distance, the ISWI remodeling complex promotes the formation of nucleosomes arranged on beads-on-a-string structures (Ito et al., 1997; Varga-Weisz et al., 1997; Corona et al., 1999; Clapier et al., 2017).

#### 3.2.1 Structure and composition

Structurally, the ATPase domain of the ISWI subfamily is separated by a small insertion sequence and flanked by two domains, the autoinhibitory N-terminal (AutoN) and the negative regulator of coupling (NegC), which function as the regulators of ATPase activity (Figure 1B) (Corona and Tamkun, 2004; Yadon and Tsukiyama, 2011; Clapier and Cairns, 2012). The C-terminal HSS domain of ISWI remodelers binds to the unmodified histone H3 tail and the linker DNA, flanking the nucleosome (Grüne et al., 2003; Boyer et al., 2004; Dang and Bartholomew, 2007; Clapier et al., 2017).

ATP-utilising chromatin assembly and remodeling factor (ACF) complex catalyzes the relaxation of the chromatin, aiding histone deposition, and the chromatin accessibility complex (CHRAC) allows nucleosome sliding and assembly on the regions with nonhomologous end-joining repair of double-strand breaks (Kukimoto et al., 2004; Lan et al., 2010). While these subfamilies regulate chromatin accessibility and thus repress transcription, an ISWI subset named the NURF complex may, in contrast, promote transcription (Xiao et al., 2001; Hochheimer et al., 2002; Clapier and Cairns, 2009). These dual roles may be associated with their differences in the activity of mechanistic sliding. Hence, the ISWI subfamily may act either as an activator or repressor during transcription, depending on the availability of other factors (Narlikar et al., 2002; Erdel et al., 2010).

In total, there are 16 ISWI subfamily complexes identified in mammals. Each complex of the ISWI subfamily is composed of one ATPase subunit, either SMARCA5 or SMARCA1, and other noncatalytic subunit(s) (Figures 2, 3B). For instance, the combination of the ATPase subunit with Bromodomain Adjacent to Zinc Finger Domain protein BAZ2B, will constitute either BRF1 (B-related factor 1) or BRF5 remodeling complexes, depending on whether the ATPase involved is SMARCA1 or SMARCA5, respectively. An exception is the NURF1 complex, which is composed of SMARCA1 with BPTF (Bromodomain PHD finger Transcription Factor) and additional RBBP4 and RBBP7 subunits, compared to the NURF5 (NUcleosome Remodeling Factor) counterpart, which only consists of the combination of SMARCA5 and BPTF.

#### 3.2.2 Role in breast cancer

ISWI remodeling complexes have multifaceted functions as they are known to promote the transcription of oncogenes (Table 1), but some of their members are also implicated in tumor suppression. Genetic alterations of ISWI members are commonly reported in many types of cancers, and they also correlate with poor prognosis of breast cancer patients (Pérez-Pena et al., 2019; Li et al., 2021), as ISWI ATPases interact with numerous DNA-binding factors and cofactors involved in malignant cell transformation and progression (Mayes et al., 2017; Koedoot et al., 2019).

Previous studies have shown that a high level of BAZ1A in HER2+ breast tumors is associated with poor overall survival and relapse-free survival, and amplification of Bromodomain PHD Finger Transcription Factor (BPTF) in breast tumors is linked to short metastasis-free survival (Pérez-Pena et al., 2019; Li et al., 2021). Overexpression of *SMARCA5* is also frequently seen in breast cancer, which is positively correlated with the stages of tumor, node and metastasis, and poor overall survival (Jin et al., 2015). SMARCA1 plays a vital role in maintaining cell survival and cell cycle progression, as inhibition of SMARCA1 leads to the upregulation of Apoptotic Protease Activating Factor 1 (*APAF1*) and thus increased activity of caspase 9 in primary breast cancers (Ye et al., 2009; Li et al., 2021). SMARCA5 may be responsible for the transition from G1 to S phase in cell proliferation and for accommodating the Matrix Metallopeptidase 2 (MMP2)-mediated invasion (Jin et al., 2015). However, according to a study by Dai et al., SMARCA1 downregulation is associated with poorer prognosis in breast cancer patients, which warrants further investigation into its role in breast cancer prognosis (Ye et al., 2009; Dai et al., 2022). Gene expression analysis by Li et al. showed that SMARCA1 is downregulated while SMARCA5 is upregulated in invasive breast carcinoma, suggesting opposing roles of these proteins (Li et al., 2021). However, other studies demonstrated increased expression of both proteins in the same context (Jin et al., 2015). Hence, a further study confirming their expression levels and functions in various subtypes of breast cancer is needed, as the balanced expression of SMARCA5 and SMARCA1 may be of interest for therapeutic purposes.

Another member of the ISWI subfamily implicated in breast cancer progression is the Remodeling and Spacing Factor (RSF) complex, which consists of a chaperone nuclear protein and SMARCA5 or SMARCA1. The overexpression and amplification of *RSF1* are reported in many cancers, including breast cancer (Figure 4), and are associated with poor overall survival, advanced clinical features, and drug resistance (Li et al., 2021). However, RSF1’s role in malignant transformation is associated with p53 expression. In normal cells, RSF1 upregulation induces Ataxia Telangiectasia Mutant (ATM)/p53-dependent DNA damage response, eventually leading to growth arrest and apoptosis. Thus, mutation or inactivation of p53 compromises the growth-inhibitory effects of RSF1 and is paired with the overexpression of SMARCA5, eventually promoting cell proliferation (Sheu et al., 2010; Kshirsagar et al., 2012). This mechanism might provide a selective advantage for p53-mutated cells, allowing RSF1 to act as a driver gene in this context (Sehdev et al., 2010; Sheu et al., 2013; Li et al., 2021).

The components of ACF and CHRAC complexes, including BAZ1A, CHRAC1, and POLE3, are also upregulated in breast cancers (Li et al., 2021). However, BAZ1A is associated with cellular senescence by regulating Mothers Against Decapentaplegic homolog 3 (*SMAD3*) and *p21* in tumor cells (Ito et al., 1999; Li et al., 2019). As for CHRAC complexes, the *CHRAC1* gene is amplified on chromosome 8q24.3 and it is confirmed to be a driver gene that promotes the proliferation and clonal survival of breast cancer cells (Mahmood et al., 2014). Like its homologous counterpart, *BAZ1B* is overexpressed and amplified in breast cancer (Figure 4). It promotes the expression of the Cytochrome P450 Family 19 Subfamily A Member 1 (*CYP19A1*) gene, which encodes the aromatase enzyme that produce estrogens from androgens (Lundqvist et al., 2018; Li et al., 2021). Aromatase inhibitors, such as Letrozole, Anastrozole, and Exemestane, reversibly bind to aromatase to prevent androgens from binding (Ghodsi and Hemmateenejad, 2016; Bhatia and Thareja, 2024). However, the upregulation of *BAZ1B* may lead to the deregulated expression of *CYP19A1* and ER-α coding gene, *ESR1*, reducing the efficacy of aromatase inhibitors. Interestingly, vitamin D analogs can be introduced to dissociate BAZ1B from the *CYP19A1* promoter and to improve the efficacy of aromatase inhibitors. This instance highlights the feasibility of targeting BAZ1B indirectly *via* vitamin D analog modulation (Lundqvist et al., 2013; Lundqvist et al., 2018).

Another documented ISWI subfamily member implicated in breast cancer progression is the NURF complex with BPTF and RBBP4/7 subunits. *BPTF* is known to be frequently amplified in breast cancer (Figure 4) (Li et al., 2021). High BPTF copy number is significantly associated with advanced tumor grade in ER+ and TN breast cancers. BPTF expression may also be responsible for promoting proliferation and controlling apoptosis, as evidenced in an *in vivo* TNBC study (Bezrookove et al., 2022). According to the study by Li et al., the expression levels of several ISWI subfamily members, including NURF complex, are strongly associated with immune checkpoint activation and/or tumor-infiltrating immune cell ratio. In breast cancer, the BPTF/NURF complex may promote the immune escape of cancer cells (Landry et al., 2011). Knockout of *BPTF* in mouse breast cancer models promotes the expression of immunoproteasome subunits Psmb8 and Psmb9 and antigen transporters Tap1 and Tap2, therefore resulting in enhanced antigenicity and T-cell cytotoxicity. Additionally, BPTF-deficient tumor cells may exhibit increased perforin, granzyme, and IFN-g due to enhanced T-cell cytotoxicity, which may induce Janus Kinase/Signal Transducers and Activators of Transcription (JAK/STAT) and Fas Cell Surface Death Receptor/Tumor Necrosis Factor-Related Apoptosis-Inducing Ligand (Fas/TRAIL) pathways (Mayes et al., 2016; Mayes et al., 2017). In breast cancer cell lines, BPTF can also inhibit the antitumor activity of NK cells by reducing the cell surface heparan sulfate proteoglycan and natural cytotoxicity receptor co-ligand abundance (Mayes et al., 2017).

Due to the involvement of RBBP4/7 in several multi-protein transcription complexes other than ISWI, there has been no exact conclusion as to whether RBBP4 and RBBP7 execute these functions in an ISWI or non-ISWI-dependent manner in breast cancer (Abbey et al., 2018; Moody et al., 2018; Glancy et al., 2021). However, in other cancers, such as gastric and liver, RBBP4 is recruited as a part of the NURF complex to promote transcription of certain factors [e.g., SRY-Box Transcription Factor 2 (SOX2) and Octamer-Binding Transcription Factor 4 (OCT4)] that drive tumor progression (Zhu et al., 2015; Ding et al., 2019; Cai et al., 2023). In breast cancer, RBBP4/7 are known to function as part of HDAC complexes. RBBP7 is documented for its role in metastasis by binding to EMT-related genes in several cancers (Li et al., 2010; Li et al., 2016; Wang J. et al., 2020; Li et al., 2021). Interestingly, the RBBP4-containing complex is also involved in drug resistance (Li et al., 2021). In TNBC, B-cell CLL/lymphoma-11A (BCL11A) is a transcription factor that contributes to maintaining the chemo-resistant breast cancer stem cell population through its interaction with RBBP4. Inhibition of RBBP4-BCL11A complex formation by BCL11A peptide inhibitor may decrease aldehyde dehydrogenase-positive breast cancer stem cells; hence, targeting interactions between RBBP4 and oncogenic transcription factors may provide opportunities for intervention (Moody et al., 2018).

Additionally, another ISWI family member, BRF1/5 complexes, are formed by the interaction of BAZ2B with SMARCA1/5 [!!! INVALID CITATION !!! (Oppikofer et al., 2017)]. *BAZ2B* is reported to be downregulated in primary breast cancer, but its role in cancer progression is yet to be elucidated (Li et al., 2021). CECR2, which is a subunit of the CECR2-containing Remodeling Factor (CERF) complex, is also implicated in breast cancer metastasis (Zhang M. et al., 2022). Upregulation of *CECR2* in breast cancer metastasis is also attributed to the modulation of tumor immunity by promoting M2 macrophage polarization to create an immunosuppressive environment. This is achieved by forming a complex with p65 through its bromodomain, which can subsequently activate the expression of NF-kB target genes such as Colony Stimulating Factor-1 (*CSF1*) and C-X-C Motif Chemokine Ligand 1 (*CXCL1*) that mediate macrophage-mediated immune suppression upon metastasis (Zhang M. et al., 2022).

### 3.3 CHD subfamily

The remodelers of the CHD subfamily are structurally similar to those of the ISWI subfamily, except that they have the tandem chromodomains. CHD remodeling factors differ in function depending on the chromodomain diversity and perform all three general remodeling processes (Lusser et al., 2005; Denslow and Wade, 2007; Konev et al., 2007; Clapier and Cairns, 2009; Kunert and Brehm, 2009; Murawska and Brehm, 2011). However, they are mainly implicated in nucleosome assembly and repression of transcription primarily through involvement as part of the Mi-2/nucleosome remodeling and deacetylase (NuRD) complexes, which contain histone deacetylases (HDAC1/2) and methyl CpG-binding domain (MBD) proteins (Figure 2) (Denslow and Wade, 2007; Clapier and Cairns, 2009; Allen et al., 2013). Predominantly, members of the CHD subfamily are associated with tumor suppression, however, some members, such as CHD1 and 8, are reported to exhibit both tumor-suppressive and pro-oncogenic functions depending on the context (Tan et al., 2014; Yu et al., 2015).

#### 3.3.1 Structure and composition

CHD family proteins are structurally similar to those of the ISWI family, except for the tandem chromodomains in the amino terminus and they only possess the NegC regulator, followed by DNA-binding domain (DBD) with only the SANT and SLIDE domain instead of the HSS domain (Figure 1B) (Tran et al., 2000; Kunert and Brehm, 2009; Hauk et al., 2010; Ryan et al., 2011). CHD proteins are further divided into three subfamilies, depending on the presence of their unique domains (Figure 3C) (Mills, 2017). For example, Subfamily I (CHD1, CHD2) possess SNF2 domains homologous to CHD1 proteins of other organisms (such as mouse Chd, which has AT-rich DNA binding domain), Subfamily II (CHD3, CHD4, CHD5) possess dual plant homeodomains (PHDs) which are known to bind to histone methylation, and Subfamily III (CHD6, CHD7, CHD8, CHD9) has domains such as Brahma and Kismet (BRK), PHD Zinc-finger like, SANT-like, CR, and DNA-binding domains (Delmas et al., 1993; Marfella and Imbalzano, 2007; Mills, 2017).

#### 3.3.2 Role in breast cancer

Since the CHD proteins control fundamental biological processes by altering chromatin compaction and regulating access to DNA, they are crucial in regulating cancer progression (Table 1). Their genes are heavily altered in around 30 types of cancers (Dahiya et al., 2019). For example, CHD subfamily I affects cancer invasion, metastasis and overall survival, while subfamily II plays a central role in cancer progression by promoting EMT and metastasis (Table 1). Although subfamily III is not strongly associated with malignancy, its deregulation is reported to modulate cancer-related pathways and survival through its upstream factors (Mills, 2017; Dahiya et al., 2019).

CHD1 is a member of the CHD subfamily I that is most associated with breast cancer pathogenesis (Mills, 2017). Estrogen inhibits the expression of microRNAs that target and degrade CHD1 transcription, thereby promoting cancer cell proliferation (Tan et al., 2014; Mills, 2017). CHD1 also increases the proliferative effect of estrogen *via* interaction with c-MYC However, inactivating mutations in *CHD1* are observed in breast cancers (Curtis et al., 2012; Weinstein et al., 2013). CHD2 has been implicated in tumor suppression, although this correlation still needs further exploration. Russo et al. proposed that CHD2 transcription induced by human chorionic gonadotropin during pregnancy may prevent breast cancer (Russo and Russo, 2012; Mills, 2017). The role of CHD2 in breast cancer progression might be more apparent in the presence of p53 heterozygosity, such as in Li-Fraumeni syndrome. This is based on the crucial function of *Chd2* in maintaining development, as *Chd2* is a candidate gene target of breast cancer genetic susceptibility gene *Mtsm1* (Marfella et al., 2006; Koch et al., 2007; Mills, 2017).

CHD subfamily II acts as a modulator of cellular proliferation and cell cycle progression (Bagchi et al., 2007; Mills, 2017) and is implicated in various processes in breast cancers, including chemoresistance, EMT, metastasis, *etc.* (Wang et al., 2011; Wu et al., 2012; Mills, 2017). CHD5 is the most well-studied among the subfamily members for its role in breast cancer. CHD5 loss-of-function or inactivation due to promoter hypermethylation, deletions and/or mutations has been reported in breast cancer pathogenesis (Bagchi et al., 2007; Mulero-Navarro and Esteller, 2008; Wu et al., 2012). In mouse models, its tumor-suppressor function is achieved by activating the cell cycle inhibitor gene, Cyclin Dependent Kinase Inhibitor 2A *(Cdkn2a)*, which encodes the two proteins *p16Ink4a* and *p19Arf* (Bagchi and Mills, 2008). Moreover, CHD5 represses the expression of WEE1, which is a mitotic checkpoint gene (Quan et al., 2014). Furthermore, Chd5, as part of the Trithorax complex, modulates the activity of the polycomb repressive group complex (PcG) and inhibits the expression of the oncoprotein subunit Bmi (Paul et al., 2013; Xie et al., 2015). CHD5, as a component of the NURD complex, also works in parallel with other NURD complex components containing histone deacetylase activity, which may further explain its role in tumor suppression (Quan et al., 2014; Quan and Yusufzai, 2014; Kolla et al., 2015). In mouse models, Chd5 functions functions as a dose-dependent tumor suppressor, as its heterozygous loss induces immortalization and tumorigenesis, while having three copies leads to senescence, apoptosis, and perinatal lethality (Bagchi et al., 2007; Mills, 2017). Overall, these findings suggest that *CHD5* expression might correlate directly with breast cancer survival. (Wu et al., 2012).

Akin to CHD5, CHD3 and CHD4 are components of the NURD complex, whose roles are implicated in transcription, proliferation, and DNA damage repair (O’Shaughnessy and Hendrich, 2013; Mills, 2017). While CHD3 is considered as an oncogene and CHD4 a tumor suppressor, recent evidence demonstrates the role of CHD4 as an oncogene in breast cancers, as its amplifications are more common (Figure 4). CHD4 regulates downstream pathways for cellular proliferation and aggressiveness through transcriptional mechanisms while maintaining genomic stability through non-transcriptional pathways (Hou et al., 2017; Luo et al., 2018; Ou-Yang et al., 2019; Wang Y. et al., 2020). Silencing *CHD4* causes an arrest in the G0 phase, significantly reducing DNA synthesis and upregulation of p21^WAF1,^ which is a cell cycle inhibitor (D'Alesio et al., 2016). Cells deficient in CHD4 show an increase in p21 expression in BRCA-proficient cell lines *via* inhibition of HDAC1 recruitment on the *p21* promoter (Hou et al., 2017). Depletion of *CHD4* significantly decreases cell proliferation and migration in HER2-positive and triple-negative breast cancer cell lines, leading to a reduction in tumor mass in luminal B and HER2-ortholog-activated triple-negative PDX and transgenic mouse models (D'Alesio et al., 2016; D'Alesio et al., 2019). The regulation of cell proliferation in HER2-positive breast cancer cell line with CHD4 depletion is explained by the observed increase in HER2 Tyr-1248 phosphorylation and subsequent inhibition of the downstream HER2/PI3K/AKT/ERK signaling cascade. *CHD4* positively correlates with cell motility and mortality, and its silencing triggers hyperacetylation of histone H3 on the E-cadherin promoter, further reducing migration and invasiveness in TNBC and non-TNBC cell lines (Luo et al., 2018). Furthermore, CHD4 regulates these aggressive properties through direct control on the expression of β1-integrin and p21 in TNBCs (Hou et al., 2017; Luo et al., 2018; Ou-Yang et al., 2019). Upon knockdown, reduction in the expression of EMT markers such as *Vimentin*, *β-catenin* and *SNAI1* was observed (Ou-Yang et al., 2019).

Moreover, CHD4 loss may promote dysregulation in autophagy, which is one of the underlying causes of cancer (Wang et al., 2013; Wei et al., 2015; D'Alesio et al., 2019). Autophagy is marked by changes in microtubule-associated protein 1A/1B-light chain 3 (LC3) levels, a protein involved in autophagosomal membrane formation and degradation (Kabeya et al., 2000; Kabeya et al., 2004). The ratio of cytosolic LC3-I level and membrane-bound LC3-II level can be used to estimate autophagic activity (Tanida et al., 2008). During the process of autophagy, an autophagy adaptor protein, p62, binds to LC3 on the autophagosome and to ubiquitinated proteins, promoting subsequent degradation (Lippai and Lőw, 2014; Liu et al., 2016). CHD4 depletion causes accumulation of p62 and an increase in the LC3 II/I ratio, suggesting a block in late-stage autophagy in HER2-positive breast cancer cells, further contributing to growth arrest in cancer cells upon CHD4 loss (D’Alesio et al., 2017; D'Alesio et al., 2019).

Even though CHD subfamily III is shown to be dysregulated in cancer, it is more implicated in neurological and developmental disorders (Ronan et al., 2013). CHD8 is a member of subfamily III with the strongest link to breast cancer. CHD8 mediates estrogen and cyclin D1-mediated recruitment of E2F1 to the promoter of cyclin E2, facilitating the proliferative effect of estrogen (Caldon et al., 2009). However, CHD8 is one of the top tumor suppressor genes, mutated in breast cancer (Figure 4) (Pongor et al., 2015). Interestingly, CHD8 is known to influence progestin-dependent gene regulation, and it also interacts with the SWI/SNF complex, where depletion of its ATPase subunits inhibits CHD8 recruitment (Ceballos-Chávez et al., 2015). *CHD7* is the most amplified CHD protein, represented in around 11% breast cancer patients (Figure 4) and its amplifications are more prevalent in aggressive breast cancer subtypes, correlating with high tumor grade and poor prognosis. This association may support the findings from *CHD7* knockdown models in triple-negative breast cancer cell lines showing inhibition of cell proliferation and decreased gene expression of several CHD7 targets, including the *NRAS* oncogene (Chu et al., 2017). *CHD3* and *CHD9* are the most deleted CHD genes in breast cancer (Figure 4), with 60% and 55% of breast cancer patients showing heterozygous loss, respectively (Chu et al., 2017). However, their role in cancer progression is still obscure to date.

### 3.4 INO80 subfamily

The most prominent function of the INO80 subfamily lies in histone editing, in which a particular histone in a nucleosome is removed in a replication-independent manner and replaced with either a canonical or variant histone (Figure 1) (Clapier et al., 2017). INO80 complex regulates chromatin structure through mobilizing mononucleosomes in an ATP-dependent manner in two different ways (Bao and Shen, 2007; Chen et al., 2013). The first is by catalyzing the sliding of nucleosomes, while the second is by catalyzing the replacement of histone H2A-H2B dimers with H2AZ-H2B dimers in nucleosomes (Shen et al., 2000; Jin et al., 2005; Papamichos-Chronakis et al., 2011; Chen et al., 2013). While H2AZ is associated with active transcription due to its role in the recruitment of transcription factors, especially on enhancers, its removal from promoters is also required for gene activation (Santisteban et al., 2000; Dhillon et al., 2006; Brahma et al., 2017). It is also implicated in homologous recombination following DNA damage and the prevention of non-coding transcription (van Attikum et al., 2004; Conaway and Conaway, 2009; Xue et al., 2015; Brahma et al., 2017). The functions of two other complexes, however, are slightly different than the INO80 complex, in which the SRCAP complex replaces canonical H2A-H2B dimers with the H2A.Z variant similar to the INO80 complex, while the p400/TIP60 complex replaces H3.1 histone with variant H3.3 (Mizuguchi et al., 2004; Papamichos-Chronakis et al., 2011; Pradhan et al., 2016; Clapier et al., 2017).

#### 3.4.1 Structure and composition

One prominent feature of INO80 subfamily is that these proteins possess a long insertion of more than 1,000 amino acids that splits the ATPase domain, which binds a hetero-hexameric ring of helicase-related (AAA-ATPase) ruvB-like proteins (Rvb1/TIP49 (TATA-binding-protein interacting protein 49) and Rvb2/TIP48 in humans). Another property shared by the members of this subfamily is the presence of the HSA domain which recruits actin and ARP (Figures 1B, 2, 3D) (Szerlong et al., 2008; Clapier et al., 2017). The three-member complexes of this subfamily are the INO80, SNF2-Related CREB-Binding Protein (CBP) Activator Protein (SRCAP) complex, and the p400/TIP60 complexes (Figure 3D). Each subfamily complex has integral protein(s) that act as the scaffold for other subunits to interact with (Mayes et al., 2014; Dijkwel and Tremethick, 2022). The INO80 complex scaffold is structurally divided into three modules that each interact with a domain of the primary INO80 scaffold (Chen L. et al., 2011). This includes one N-terminal interacting module consisting of nuclear factors related to kB (NFRkB), Amida, Microspherule protein 1 (MCRS1**)**, Ubiquitin C-Terminal Hydrolase (UCH37), INO80D, and INO80E; the HSA-interacting module that is composed of actin-related proteins ARP4, ARP8, and Kruppel family zinc finger transcription factor Yin Yang 1 (YY1**)**; and SNF2-ATPase interacting module which contains TIP49a, TIP49b, Inositol-eighty subunit 2 (IES2) (Chen et al., 2013). The SRCAP complex is composed of SRCAP as the primary scaffold that interacts with two modules, the ARP module and the motor module. The motor module of the SRCAP complex consists of YL1, ACTin Related protein 6 (ACTR6), TIP49a/b hexamers, and Zinc finger HIT-type containing 1 (ZnHIT), while the regulatory ARP module comprises b-actin, BAF53a, DNA Methyltransferase 1 Associated Protein 1 (DMAP1), and Glioma Amplified Sequence 41 (GAS41) (Krogan et al., 2003; Kobor et al., 2004; Mizuguchi et al., 2004).

The ARP module is shared between the SRCAP and TIP60/p400 complex, while the motor module of the TIP60/p400 complex possesses only YL1 and TIP49a/b hexamers as its subunit (Jacquet et al., 2016; Feng et al., 2018). The TIP60/p400 complex has Malignant Brain Tumor Domain-containing protein 1 (MBTD1) as an additional subunit for its ARP module and three other modules, including the Trimer Independent of Nucleosomal Acetyltransferase of H4 (NuA4) for Transcription Interactions with Nucleosomes (TINTIN) module for histone marker reading function, histone acetyltransferase (HAT) module for catalytic function, and Transformation/Transcription Domain Associated Protein (TRRAP) module for transcription activator binding function. The TINTIN module consists of several subunits, such as MRG domain Binding Protein (MRGBP), Mortality Factor 4 Related Gene on chromosomes 15 (MRG15), and BRD8. In contrast, the Histone acetyltransferase (HAT) module is composed of Enhancer of Polycomb Homolog 1 (EPC1), TIP60, INhibitor of Growth family member 3 (ING3), and Esa1-Associated Factor 6 (EAF6) (Jacquet et al., 2016). The TRRAP module is composed of only TRRAP as a large, single subunit that is tethered to the SANT domain of P400 (Auger et al., 2008).

#### 3.4.2 Role in breast cancer

Although there are very few documented studies on the INO80 subfamily in the context of breast cancer, there has been a growing interest in researching this chromatin remodeling complex due to its unique function in editing nucleosome composition and histone specialization, which changes nucleosome stability (Clapier, 2021).

INO80 complex aberrations are associated with cancer progression, as their binding to enhancers is crucial in mediating oncogenic gene expression (Min et al., 2013; Wang et al., 2014; Runge et al., 2018; Thang et al., 2023). In the TCGA breast cancer atlas cohort, the subunits of the INO80 complex are frequently amplified, accounting for alterations present in around 5% of overall breast cancer cases (Figure 4) (Thang et al., 2023). However, when characterized under PAM50-based subtyping, *INO80* expression is significantly downregulated in basal-type breast cancer (Thang et al., 2023) Importantly, INO80 functions as a mediator of dynamic replacement of an active enhancer-based histone variant, H2A.Z, and thus as a critical regulator of enhancers close to E2 target genes such as *GREB1* and *TFF1*, contributing to breast cancer progression (García-Pedrero et al., 2006; Papamichos-Chronakis et al., 2011; Segala et al., 2016; Thang et al., 2023). Thang et al. further explained the relationship between INO80 and ER through Weighted Gene Co-expression Network Analysis (WGCNA), which indicated the existence of networks between INO80 and a subset of luminal breast cancer biomarkers, including *ESR1*, *FOXA1*, and other ER target genes such as *GATA3, TFF1,* and *AR* (Tozlu et al., 2006; Chen Y. et al., 2011; Hurtado et al., 2011; Thang et al., 2023). However, lower expression of *INO80* is associated with reduced overall survival rate, distant metastasis-free survival, and recurrence-free survival in breast cancer patients (Thang et al., 2023). Low *INO80* copy number status is also associated with an increased risk in TNBC patients compared to luminal and HER2+ patients. While INO80 affects the ductal morphogenesis and differentiation of the mammary gland, knockout of *INO80* alone is not enough to cause cancerous changes in mouse mammary gland models through mechanisms yet to be understood.

Ubiquitin Carboxyl-terminal Hydrolase 35 (UCH37), encoded by *UCHL5*, is heavily amplified (Figure 4) and is associated with poor outcomes in breast cancer patients (Curtis et al., 2012; Weinstein et al., 2013). UCH37 is a deubiquitinase oncoprotein in the INO80 chromatin remodeling complex (VanderLinden et al., 2015). This protein can activate deubiquitination of E2 promoter binding factor F1 (E2F1), which subsequently enhances the transcription and increases the proliferative activity of E2F1 target genes (Mahanic et al., 2015). Moreover, *UCHL5* amplification is associated with higher activity of the TGF-β signaling pathway through the formation of Smad ubiquitination regulatory factor 2 (Smurf2) – Smad7 complex. This affects the expression of subsequent TGF-β downstream targets such as *MMP-2* and *PAI-1* that are crucial in regulating tumor migration and invasion (Wicks et al., 2005; Wicks et al., 2006; Cutts et al., 2011).

Besides INO80, other subfamily members, including SRCAP and p400/Tip60 complexes, also regulate H2A.Z deposition into chromatin (Ruhl et al., 2006; Svotelis et al., 2010). Moreover, the p400/Tip60 complex is known to indirectly affect cell proliferation by regulating the expression of several transcription factors, such as *E2F1*, *p53*, *KAI1*, and *Myc* (Taubert et al., 2004; Kim et al., 2005; Samuelson et al., 2005; Nagl et al., 2007; Svotelis et al., 2010). Another study by Cao et al. on a long non-coding RNA, LINC00665 in breast cancer cell lines shows that it can inhibit miRNA- 641, which affects SRCAP translation by binding to its 3′UTR region. This loss of SRCAP inhibition results in proliferation and invasion of breast cancer cells, suggesting its role as an oncogene (Cao et al., 2022). This suggests that the regulation of p400/Tip60 and SRCAP complexes is related to breast cancer progression, but their exact function needs further exploration. YL1, encoded by the vacuolar protein sorting 72 homologs (*VPS72*) gene, is a subunit shared by the SRCAP and TIP60/p400 complex and is frequently amplified in many cancers, including breast (Figure 4) and melanoma. Given the prevalent alteration in breast cancer, this prompts further research on the possibility of utilizing *VPS72* expression as a prognostic indicator in breast cancer.

## 4 Potential therapeutic approaches

### 4.1 Targeting SWI/SNF family members

**FIGURE 5:**
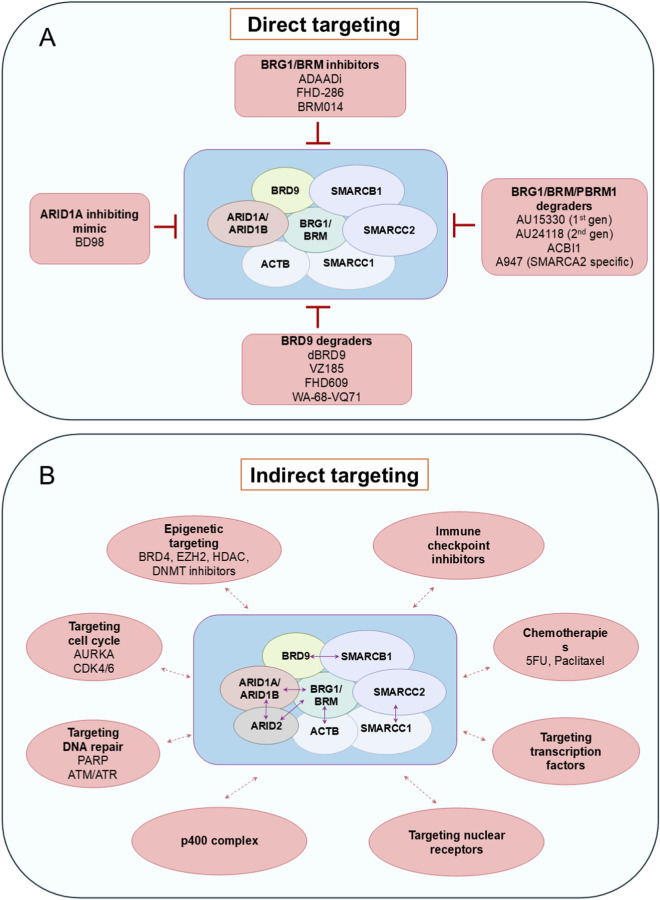
Targeting SWI/SNF complex using direct and indirect strategies. **(A)** Inhibitors and degraders (PROTACs) which were developed to target SWI/SNF function. gen–generation. **(B)** Synthetic lethality interactions with SWI/SNF complex subfamilies. Blue double-headed arrows represent synthetic lethality interactions within the complex subfamilies. Red double-headed arrows represent synthetic lethality interactions with other complexes or pathways.

As described in this review, chromatin remodelers are vital to several essential biological pathways and, therefore, pose a challenge to treat without off-target effects. Due to their significant involvement in breast cancers, their subunits can act as attractive therapeutic targets, however, they are poorly investigated in breast cancers and their association with other chromatin-associated complexes may complicate the effects. Several efforts have been made to exploit the vulnerabilities of each chromatin remodeling subfamily in other cancers, which are explained below as potential therapeutic approaches to target breast cancers. Strategies targeting these complexes can generally be divided into two categories: direct and indirect targeting (Figures 5A,B) (Zhang and Li, 2022).

#### 4.1.1 Direct targeting approaches

Targeted therapies can be directed against the exact aberrant chromatin remodelers that are altered in various types of cancer (Zhang and Li, 2022). These may work by inhibiting the ATPase catalytic subunit of the remodeling complex (for example, competitive ATP inhibitors or allosteric agents) or by disrupting the protein-protein interactions of the catalytic subunits. Presence of ATPase domains in the chromatin remodeling proteins is therapeutically beneficial due to their druggable enzymatic pockets, offering highly selective inhibitors.

For example, the ATPase activity of SWI/SNF members BRG1 and BRM can be blocked by dual allosteric small-molecule inhibitors, BRM014 or orally bioavailable inhibitor FHD-286 (Figure 5A) in BRG1-deficient lung cancer, acute myeloid leukaemia and uveal melanoma models (Papillon et al., 2018; Rago et al., 2020; Vaswani et al., 2025). Additionally, inhibitors of the SWI/SNF bromodomain, like PFI-3, have little effect as a single agent, but they can sensitise cancer cells to DNA damage by doxorubicin (Lee et al., 2021). Furthermore, Active DNA-dependent ATPase A Domain inhibitor (ADAADi) was the first SWI/SNF catalytic activity inhibitor, discovered from the byproduct of the activity of aminoglycoside phosphotransferases (Figure 5A). This exerts its function as a competitive inhibitor against DNA-dependent ATPase domains of SWI/SNF (Muthuswami et al., 2000; Dutta et al., 2012). However, its specificity towards SWI/SNF ATPase is unclear, and although it was able to sensitize breast cancer cells to chemotherapy agents, variable responses were observed in different cell lines (Rakesh et al., 2021; Dreier et al., 2024).

Importantly, subunits other than the catalytic and bromodomains of the chromatin remodeling complex, especially the SWI/SNF complex, possess virtually flat functional interfaces, due to the lack of hydrophobic ligand binding pockets, hence exhibiting a weak possibility for ligand interaction (Sammak and Zinzalla, 2015; Dang et al., 2017). This makes them undruggable with conventional approaches (Ye et al., 2019). Hence, a targeted protein degradation strategy has been pursued recently to answer this problem. This consists of a proteolysis-targeting chimera (PROTAC) with an ubiquitin-proteasome system (UPS) that precisely targets a protein by bringing an E3 ubiquitin ligase to the protein of interest and induces its ubiquitylation and degradation (Schneider et al., 2021; Békés et al., 2022). Some examples of targeted protein degraders are BRD9-directed degrader (dBRD9); VZ185, which targets BRD9 and BRD7; AU15330, AU24118, and ACBI1, which degrade SMARCA4, SMARCA2 and PBRM1 and A947, as a SMARCA2-selective degrader (Figure 5A) (Remillard et al., 2017; Zoppi et al., 2018; Farnaby et al., 2019). PROTAC-based inhibitors against SMARCA4, SMARCA2, and PBRM1 exert genomic effects by disrupting SWI/SNF interactions with chromatin (Singh et al., 2007; Filippakopoulos et al., 2012). The relevant example for this class is AU-15330, which degrades by linking the bromodomain ligand of BRG1/BRM/PBRM1 to the ligand of von Hippel-Lindau (VHL) E3 ubiquitin ligase. AU15330 efficiently targets nuclear receptor-positive cancers, such as AR-dependent prostate cancers, by reducing DNA accessibility at enhancer elements, disrupting enhancer-promoter loops, and ultimately suppressing oncogenic gene expression (Xiao et al., 2022).

#### 4.1.2 Indirect targeting approaches

Due to the complexities of direct targeting of SWI/SNF components, sometimes indirect targeting is a valid option. Indirect targeting therapies take advantage of mutations already present in cancer cells, rendering them sensitive to the inhibition of other proteins through a mechanism known as synthetic lethality. Cancers with loss-of-function mutations in tumor suppressors may benefit more from indirect targeted therapies, where synthetic lethal interactions can be exploited (Figure 5B) (Nijman, 2011; Wanior et al., 2021). The most common example of these interactions is provided by the combination of mutually exclusive gene paralogues, such as in cancers with *ARID1A* mutation that require ARID1B for survival or tumors with *BRG1* deficiency, which are sensitive to SMARCA2 depletion (Kaelin, 2009; Helming et al., 2014; Hoffman et al., 2014). Hence, selective degradation of the SWI/SNF ATPase SMARCA2 by PROTACs YDR1 or YD54 was found to provide better sensitivity in SMARCA4 mutant xenografted lung cancer cells (Kotagiri et al., 2025). Other known synthetic lethal interactions are BRD9/SMARCB1, SMARCC1/SMARCC2, BRG1/ACTB, BRG1/ARID2 and BRG1/BRM/ARID1A with p400 complex (Figure 5B) (Michel et al., 2018; Schick et al., 2019; Martin et al., 2023). Synthetic lethality can be induced in several ways, such as by inhibiting the DNA damage repair process in case of SWI/SNF mutant tumors. For example, *ARID1A*-deficient cancers respond to PARP inhibitors due to ARID1A’s role in DNA repair; ATR/ATM inhibitors are synergistic in cancers with ARID1A-deficiency or BAF-inhibition, and cancers with *PBRM1, BRD7,* or *BRD9* inactivation are sensitive to replication stress and can be treated with PARP and ATR inhibitors (Shen et al., 2015; Williamson et al., 2016; Hu et al., 2019; Chory et al., 2020; Zhou et al., 2020; Chabanon et al., 2021). A similar response can also be seen by targeting proliferation-related factors. For instance, CDK4/6 inhibitors can sensitise *BRG1*-defective cancer cells, especially in small cell carcinoma of the ovary hypercalcemic type (SCCOHT) and Non-small cell lung cancer (NSCLC) due to the downregulation of cyclin D1 (Xue et al., 2019a; Xue et al., 2019b). Similarly, Aurora Kinase A (AURKA) promotes cell cycle progression and can be targeted in *ARID1A*-deficient colorectal cancer cells (Wu et al., 2018).

Other indirect targeted therapies include epigenetic therapies that target post-translational modifications of histones and immunotherapy-based approaches such as immune checkpoint blockade therapies. Epigenetic drugs like histone deacetylase (HDAC), DNA methyltransferase (DNMT), and enhancer of zest homolog 2 (EZH2) inhibitors are effective in SCCOHT with *BRG1* loss-of-function, while pan-HDAC inhibitors are studied for their efficacy in treating *ARID1A-*mutated tumors (Fukumoto et al., 2018; Auguste et al., 2020). Mutations in *ARID1A/ARID1B/ARID2* render cancers more prone to immune checkpoint blockade therapies (Zhu et al., 2022). The combination of HDAC6 inhibitor and anti-PD-L1 therapy in *ARID1A*-mutated ovarian cancers showed promising antitumor effects due to improved cytotoxic T-cell activity (Fukumoto et al., 2019). Moreover, PBRM1, which plays a role in anti-tumor immune response, might be a valuable and prospective therapy target.

Despite the tumour suppressive role of ARID1A and loss of BRG1 activity in ARID1A-perturbed ER+ breast cancers (Nagarajan et al., 2020), BRG1 and BRM may present as efficient targets in other subtypes due to their crucial roles in promoting progression and mediating resistance to chemotherapy. Previous research shows that the knockout of *BRG1* increases the sensitivity to chemotherapy drugs such as 5-FU and paclitaxel (Wu et al., 2016b). *BRG1* depletion reduces the gene expression of a drug transporter, ATP-binding cassette (ABC). This eliminates transporter induction, leading to an increase in intracellular drug concentration and sensitivity to chemotherapy (Dubey et al., 2016; Wu et al., 2016b; Wu et al., 2017). Coherently, the inhibition of drug efflux due to the absence of BRG1 also decreases the ability of the cancer cells to eliminate DNA damage (Li K. et al., 2024). This highlights the importance of inhibiting BRG1 and BRM, particularly in cases such as TNBCs, which often rely on nonspecific cytotoxic drugs (Sobczak et al., 2020).

Furthermore, utilising SWI/SNF-based synthetic lethal approaches presents a severe drawback in tumours where other complex subunits present with tumor suppressive function (Narlikar et al., 2013; Zinzalla, 2016). More careful approaches are crucial to selectively target oncogenic function while leaving tumor suppression function unaffected in the SWI/SNF complex. These can include narrowing the therapeutic window or inhibiting specific protein interactions (Zinzalla, 2016). Either the intra-complex protein-protein interactions (PPI) with cytoplasmic signaling effectors or transcription factors can be inhibited. For example, the manipulation of the Armadillo repeats (ARM) domain of the ARID1B subunit may disrupt ARID1B interaction with other subunits in the SWI/SNF complex and induce synthetic lethality in ARID1A-deficient cancers (Takada et al., 2012; Yu et al., 2013). Another approach may involve inhibiting the interaction with oncogenic transcription factors (MYC, JUNB, Notch1, beta-catenin, STAT1/2/3, SMAD2/3, NF-kB, and TCF3) or tumor suppressors (pRB, p53, and BRCA1) (Zinzalla, 2016). On the other hand, identifying the exact subunit that mediates the interaction with a specific transcription factor provides another opportunity, but only a handful have been identified. ARID1A possesses the interacting protein motifs (LXXLL), which enable binding to nuclear hormone receptors. SMARCD1 and SMARCD3 have been shown to interact with transcription factors, such as p53, NF-kB, and SALL4. Hence, developing selective inhibitors to target these interactions is of great interest (Puri and Mercola, 2012; Zinzalla, 2016).

### 4.2 Targeting other chromatin remodelers

In addition to the SWI/SNF subfamily, bromodomains of the ISWI subfamilies can be targeted (Pérez-Salvia and Esteller, 2017). For example, by targeting the bromodomain, a small molecule inhibitor NVS-CECR2-1 can affect CECR2 binding to chromatin and induce apoptosis in several cancer cell lines (Pérez-Salvia and Esteller, 2017; Park et al., 2020). Considering the role of CECR2 in modulating the immune response in the breast cancer tumor microenvironment and promoting metastasis, utilizing this agent to achieve an immune-responsive tumor microenvironment might be a promising approach to counter metastatic breast cancer with upregulated CECR2. Since BPTF is known to be frequently altered in breast cancer, inhibition of the BPTF bromodomain by aryl urea-1 (AU1) is a promising strategy to target the NURF1/5 remodeling complex (Frey et al., 2017). AU1 causes G1 arrest by impeding chromatin accessibility, lowering c-MYC occupancy, and subsequently diminishing the proliferative capacity of cancer cells. Another prospective BPTF inhibitor is sanguinarine chloride, which showed a potent antiproliferative effect in pancreatic cell lines by downregulating c-Myc expression (Liang et al., 2023). Although BPTF is known for its extensive role in regulating immunity in tumor microenvironments, the extent to which AU1 inhibition affects the BPTF immune function remains to be elucidated. Another selective competitive inhibitor that targets BAZ2A/B bromodomains is GSK2801. While it has no significant tumor suppressive effect as a single agent, GSK2801 showed a strong synergistic activity along with the BET inhibitor JQ1 in several TNBC cell lines by targeting BRD2 (Bromodomain-containing protein 2), which is co-regulated along with BAZ2A/B (Bevill et al., 2019). GSK2801 displaces BRD2 at the promoters of genes regulated by ETS (E26 transformation-specific) transcription factors and at 45S ribosomal DNA promoters, which induces caspase-3 activity and PARP cleavage, leading to apoptosis of tumor cells in the 3D cultures (Bevill et al., 2019). Although the mutation frequency of BAZ2A/B is insignificant in breast cancer, this instance suggests the possibility of targeting the chromatin remodeling complexes with synergistic inhibitors to overcome the limitation of single-agent therapies.

Despite its significant alteration rate and role as a driver gene for breast cancers, CHRAC1 does not possess a bromodomain and presents a significant challenge in developing small-molecule inhibitors. However, CHRAC1 is known to potentially interact with YAP in breast cancer and exerts its tumorigenesis effect by enhancing YAP target oncogenes, which are involved in the Hippo pathway (Li S. et al., 2024). Hence, targeting the interaction between CHRAC1 and YAP can provide an essential and novel approach to breast cancer treatment strategy. RSF1 is also faced with a similar issue due to the lack of bromodomain or catalytic domains, and further study on RSF1 interaction partners in breast cancers may potentially give therapeutic benefits.

Modulating the post-translational modifications is the most feasible approach for targeting CHD subfamilies, as no specific inhibitors have been discovered to date. Synthetic lethality between CHD1 and PTEN can also be employed, as seen in the *PTEN*-deficient xenograft model (Zhao et al., 2017). Since members of the CHD subfamily usually compose or interact with larger protein complexes, inhibiting other vital interactors, i.e., HDAC inhibitors, can also disrupt the function of CHD remodelers (Xue et al., 1998; Low et al., 2016; Smith et al., 2018). Moreover, CHD3 and CHD4 are known to participate in DNA repair, and the recruitment is mediated by PARP activity (Smith et al., 2018). Hence, PARP inhibitor AG-014699 and the suberohydroxamic acid as an HDAC inhibitor have been shown to inhibit CHD4 function and tumor growth in a mouse xenograft model of EpCAM^+^ hepatocellular carcinoma (Nio et al., 2015). Lastly, E3 ubiquitin ligases, such as SCFβ-TrCP E3 ligase, which targets CHD1 and FBXW7 E3 ligase for CHD6 protein, are being developed (Zhao et al., 2017; Zhang B. et al., 2022). Development of E3 ligases for CHD7 degradation may be beneficial for breast cancer treatment (Stark et al., 2006; Wagner et al., 2011; Beltrao et al., 2012; Povlsen et al., 2012; Akimov et al., 2018; Ordureau et al., 2020; Pedersen et al., 2021).

Despite being a potential target in many cancers, inhibition of INO80 complex subunits is poorly investigated. There are very few studies on inhibiting the INO80 and SRCAP complexes, for example, using Inositol hexaphosphate (IP6) against the INO80 complex (Shen et al., 2003; Willhoft et al., 2016).

## 5 Discussion

### 5.1 Direct targeting strategies

While the direct targeted therapies offer precision and selectivity in inhibiting the proteins of interest, the complexity of the chromatin remodeling complexes may raise problems with this approach. Furthermore, the specificity of direct targeted therapies in inhibiting specific proteins may also limit their broader applicability. Patients with mutations or aberrations on the target genes that provide synthetic lethality may benefit from the above therapies, however, identifying the aberrations occurring in breast cancer patients before initiating therapies is laborious and poses financial burdens for clinical settings.

Another pitfall is the capability of these therapies to achieve context-dependent effects in different cancers. Intratumoral heterogeneity coerces more complexity by establishing different molecular subtypes; for example, in small cell lung cancers (SCLC), these signatures are driven by transcription factor, which include achaete-scute family bHLH transcription factor 1 (ASCL1), neurogenic differentiation factor 1 (NeuroD1), POU domain class 2 transcription factor 3 (POU2F3), and yes-associated protein 1 (YAP1) (Borges et al., 1997; Huang et al., 2018; Rudin et al., 2019; Duplaquet et al., 2024; He et al., 2024). Among these subtypes, POU2F3-driven SCLC (SCLC-P) exhibits SWI/SNF dependency, especially towards SWI/SNF ATPases and ncBAF complex subunits, including BRG1, SMARCD1, and BRD9 (Huang et al., 2018; Duplaquet et al., 2024; He et al., 2024). This is evidenced by strong effects on proliferation and expression of POU2F3 and its coactivators upon BRD9 degradation (using FHD-609, WA-68-VQ71, or dBRD9A) on non-neuroendocrine POU2F3+ SCLC models, and pan-SWI/SNF ATPase inhibition or degradation (using FHD-286, BRM014 and AU-15330) on all POU2F3+ cells (Figure 5A) (Duplaquet et al., 2024). Further analyses showed that the reduction in POU2F3 levels is due to the impairment of the chromatin accessibility of the POU2F3 gene. This dependency serves as the basis for utilizing the SWI/SNF ATPase inhibitor as a therapeutic agent for SCLC-P. However, other subtypes of SCLC are resistant to SWI/SNF inhibition, underscoring the importance of molecular subtype identification in cancers with diverse intratumoral heterogeneity and context-specific association of transcription factors with epigenetic modulators (Huang et al., 2018; Duplaquet et al., 2024).

While PROTACs like AU15330 provide high selectivity over ATPase subunits of SWI/SNF complex, they may not provide a therapeutic advantage in cancers where BRG1 and ARID1A are tumor suppressors, as BRG1/BRM degradation may further compromise their tumor-suppressive ability and jeopardize the cancer cells by developing other mutations due to deregulated genomic instability. Moreover, several breast cancer cell lines, such as MCF7, MDA-MB-486, and MDA-MB-231, exhibit resistance to AU15330 treatment (Xiao et al., 2022). These issues may be associated with subtype heterogeneity and genetic diversity in cancer cell line models (Navin et al., 2010; Shah et al., 2012; Yang et al., 2018). Further studies exploring subtype-specific vulnerability against SWI/SNF inhibitor agents in various breast cancer *in vivo* models may give a better understanding of context-specific effects in breast cancer treatment. To aid this strategy, an advanced molecular profiling with analyses of differential gene expression and chromatin profiling can be employed to identify whether BRG1 or any other targeted proteins of interest predominantly support oncogenic or tumor-suppressing pathways. If it primarily supports oncogenic pathways, it is safe to assume that inhibiting BRG1 would help suppress breast cancer progression. Otherwise, the inhibition of the BRG1 might be deleterious, unless the patients are presented with BRG1 or ARID1A/ARID1B mutations, which require careful monitoring of gene alterations. Moreover, due to the broad expression of SWI/SNF subunits in cells, adverse side effects such as differentiation syndrome, hyperbilirubinemia, *etc.*, were observed in patients treated with FHD-286 in phase 1 clinical trials (Dinardo et al., 2025). Hence, localizing the drug administration to cancerous tissue and minimizing exposure to normal cells by utilizing targeted delivery systems, such as with nanoparticles or antibody-based systems, can aid in reducing the adverse side effects. Combination with chemotherapeutic agents would also provide a better outcome by making the proliferating cells more vulnerable to these targeted therapies.

In addition, inadequate pharmacological properties for clinical use may also cause concerns. For example, AU24118 is more beneficial than AU15330 in treating SCLC-P xenografts NCI-H1048 and NCI-H211 due to its oral bioavailability and enhanced pharmacokinetic properties (He et al., 2024). AU24118 also downregulates POU2F3 and its coactivator, resulting in the reduction of tumor volume and weight. Other agents, such as FHD-609 and FHD-286, which act as BRD9 degraders and BRG1 inhibitors, respectively, have shown a promising increase in survival rate after 35 days of continuous treatment in the same SCLC-P xenografts (Duplaquet et al., 2024). Furthermore, these agents can be used with conventional chemotherapies for SCLC, such as cisplatin and etoposide, and have shown additive and synergistic anti-tumor effects without posing toxicity in multiple SCLC-P models (Duplaquet et al., 2024; He et al., 2024). Hence, exploring the efficacy of these improved clinical-grade inhibitors across different models and molecular contexts is of interest in addressing the intratumoral heterogeneity and resistance mechanisms in breast cancers.

While the premise of blocking BRG1 and BRM might seem prospective, ATPase-binding sites are very dynamic since they modulate intermolecular interactions during the remodeling cycle. The high conservation of ATPase domains and allosteric modulation of the pocket conformation by protein binding partners complicates drug design (Racki et al., 2014). While PROTAC is a beneficial strategy, the disruption of SWI/SNF subcomplexes through bromodomain degradation may create residual complexes without catalytic activity. These residual complexes can still activate deregulated transcription factor networks and drive oncogenic gene expression (Pan et al., 2019; Cantley et al., 2022). Lastly, the discovery of BD98, a macrolactam inhibitor, mimics the effect of ARID1A/B loss (Chory et al., 2020; Dreier et al., 2024). This agent can be best combined with ATR inhibitors for breast cancers, which can improve the efficacy of adaptive T-cell therapy by preventing differentiation (Guo et al., 2022). However, there are limited studies on this agent, and inhibitors for ATPase domains and bromodomains are still the most common approach (Dreier et al., 2024).

Despite their feasibility and potential, several challenges related to prolonged SWI/SNF inhibitor exposure demand further consideration for clinical use. This includes exploring the side effects of long-term administration, the chance of predisposing patients to secondary malignancies, the impact on SWI/SNF-maintained adult stem cells, and investigating the efficacy of combination therapies. Moreover, studies on potential drug resistance issues and biomarkers for monitoring and predicting prognosis still need to be explored (Dreier et al., 2024).

### 5.2 Indirect targeting strategies

While indirect strategies offer a more effective approach due to exemplar and well-studied synthetic lethality approaches that sensitise the cancer cells, context-specific effects must also be considered. For example, HDAC inhibition might not be a favorable therapeutic option for ARID1A-mutated ER+ breast cancers, as ARID1A-loss-induced endocrine resistance is mediated by the loss of HDAC1 activity (Nagarajan et al., 2020). Interestingly, the same study showed that the hyperacetylation and reprogrammed binding of BRD4 due to HDAC1 loss in these models can be manipulated as a therapeutic strategy using BET/HAT inhibitors. Unfortunately, BET inhibitors are not selective to BRD4 alone, and the pharmacokinetic profiles of many BET inhibitors failed tremendously in the initial phases of clinical trials due to dose-limiting toxicities such as thrombocytopenia (Doroshow et al., 2017; Sun et al., 2021; Trojer, 2022). Other strategies, such as controlled dosage of selective BRD4 targeting using PROTACs in combination with chemo- or endocrine therapies, may provide some potential benefits. Furthermore, novel vulnerabilities that align with the defined genetic defects need to be identified by applying genomic screens in an unbiased manner.

Although inhibiting the interactions of lineage-specific transcription factors with chromatin remodelers can provide ideal and specific targeting against cancers, it is significantly more difficult due to the undruggable nature of IDR regions, which interact with transcription factors (Sammak and Zinzalla, 2015). Hence, developing alternative strategies like interfering peptides against IDRs may present promising therapeutic approaches for treating breast cancers (Patil et al., 2023; Xie et al., 2025).

## 6 Conclusion and future perspectives

All subfamilies of chromatin remodeling complexes influence and regulate breast cancer aggressiveness to different extents. While there is a growing body of literature supporting their role in cancer, further studies using genetic and molecular profiling of breast tumours can delineate their tumor suppressor or coactivator activity and context-specific functions. This can reveal new avenues for developing effective therapeutic targets for breast cancer. Targeting subunits or other complexes that show synthetic lethal interactions may be feasible, which can tailor the treatment to deregulated cancer cells, overcome adverse side effects, and target resistance and relapse in breast cancer. In addition, the direct or indirect therapies targeting chromatin remodeling complexes should be tested in combination with existing systemic and immune checkpoint therapies, as this strategy might provide synergistic actions and potential benefits to cancers with genetic alterations on these complex subunits. Further exploration of transcription factor-specific associations is warranted to develop therapies in a subtype-specific manner and to establish precision medicine in relevance to complex-specific mutations. Research avenues must also explore the possibility of exploiting the chromatin remodeling complexes as measurable endpoint biomarkers for predicting therapeutic response and prognosis in breast cancer.

## Glossary

ER: Estrogen receptor
PR: Progesterone receptor
HER2: Human EGF receptor 2
TNBC: Triple negative breast cancer
SWI/SNF: SWItch/sucrose non-fermentable
ISWI: Imitation SWItch
CHD: Chromodomain helicase DNA-binding
INO80: INOsitol-requiring mutant 80
SANT: Swi3, Ada2,nuclear receptor corepressor and transcription factor IIIB
HSA: Helicase SANT-associated
HSS: HANT-SANT-SLIDE
ARP: Actin-related proteins
BAF: BRG1-associated factors
PBAF: Polybromomo-associated BAF
ncBAF: Non-canonical BAF
BRM: Brahma
BRG1: Brahma-related gene
SS18: Synovial Sarcoma Translocation Gene on Chromosome 18
SS18L1: SS18-Like 1
ACTB: Actin Beta
ACTL6A/B: Actin Like 6A/B
BCL7A/B/C: B-Cell Lymphoma 7 Protein Family Member A/B/C
ARID1A/ARID1B: AT-Rich Interactive Domain-Containing Protein 1A/1B
DPF1/2/3: Doubled PHD Fingers Family 1/2/3
PHF10: PHD Finger Protein 10
PBRM1: Polybromo 1
BRD7: Bromodomain Containing 7
BICRA: BRD4 Interacting Chromatin Remodeling Complex Associated Protein
BICRAL: BICRA Like
EREs: estrogen-responsive elements
PCAF: p300/CBP-associated factor
IDRs: intrinsically disordered regions
PIK3CA: Phosphatidylinositol-4,5-Bisphosphate 3-Kinase Catalytic Subunit Alpha
PTEN: Phosphatase and Tensin Homolog
WT: Wild-type
ChIP-seq: Chromatin Immunoprecipitation assay with sequencing
BET: Bromodomain and Extra-Terminal domain
FOXA1: Forkhead Box A1
TEAD4: TEA Domain Transcription Factor 4
GSEA: gene set enrichment analysis
TP63: Tumor Protein P63
AutoN: autoinhibitory N-terminal
NegC: negative regulator of coupling
ACF: ATP-utilising chromatin assembly and remodeling factor
CHRAC: chromatin accessibility complex
BAZ2B: Bromodomain Adjacent to Zinc Finger Domain protein
BRF1: B-related factor 1
BPTF: Bromodomain PHD finger Transcription Factor
NURF5: NUcleosome Remodeling Factor
TNM Stage: Tumor, node and metastasis stage
APAF1: Apoptotic protease activating factor 1
MMP2: Matrix metallopeptidase 2
ATM: Ataxia telangiectasia mutant
SMAD3: Mothers against decapentaplegic homolog 3
CYP19A1: Cytochrome P450 family 19 subfamily A member 1
JAK: Janus Kinase
STAT: Signal transducers and activators of transcription
Fas: Fas cell surface death receptor
TRAIL: Tumor necrosis factor-related apoptosis-inducing ligand
SOX2: SRY-Box Transcription Factor 2
10/04: Octamer-Binding Transcription Factor 4
EMT: epithelial-mesenchymal transition
BCL11A: B-cell CLL/lymphoma-11A
CERF: CECR2-containing Remodeling Factor
CSF1: Colony Stimulating Factor-1
CXCL1: C-X-C Motif Chemokine Ligand 1
DBD: DNA-binding domain
NuRD: nucleosome remodeling and deacetylase
HDAC: histone deacetylases
MBD: methyl CpG-binding domain
Cdkn2a: Cyclin Dependent Kinase Inhibitor 2A
PcG: polycomb repressive group complex
LC3: microtubule-associated protein 1A/1B-light chain 3
CBP: CREB-Binding Protein
SRCAP: SNF2-Related CBP Activator Protein
NFRkB: nuclear factors related to kB
MCRS1: Microspherule protein 1
UCH37: Ubiquitin C-Terminal Hydrolase
YY1: Yin Yang 1
IES2: Inositol-eighty subunit 2
ACTR6: ACTin Related protein 6
ZNHIT: Zinc finger HIT-type containing 1
DMAP1: DNA Methyltransferase 1 Associated Protein 1
GAS41: Glioma Amplified Sequence 41
MBTD1: Malignant Brain Tumor Domain-containing protein 1
NuA4: Nucleosomal Acetyltransferase of H4
TINTIN: Transcription Interactions with Nucleosomes
HAT: histone acetyltransferase
TRRAP: Transformation/Transcription Domain Associated Protein
MRGBP: MRG domain Binding Protein
MRG15: Mortality Factor 4 Related Gene on chromosomes 15
EPC1: Enhancer of Polycomb Homolog 1
ING3: INhibitor of Growth family member 3
EAF6: Esa1-Associated Factor 6
WGCNA: Weighted Gene Co-expression Network Analysis
E2F1: E2 promoter binding factor F1
SMURF2: Smad ubiquitination regulatory factor 2
LINC00665: Long Intergenic Non-Protein Coding RNA 665
VPS72: vacuolar protein sorting 72 homologs
ADAADi: Active DNA-dependent ATPase A Domain inhibitor
PROTAC: proteolysis-targeting chimera
UPS: ubiquitin-proteasome system
dBRD9: BRD9-directed degrader
VHL: von Hippel-Lindau
SCLC: small cell lung cancers
ASCL1: achaete-scute family bHLH transcription factor 1
NeuroD1: neurogenic differentiation factor 1
POU2F3: POU domain class 2 transcription factor 3
YAP1: yes-associated protein 1
SCLC-P: POU2F3-driven SCLC
SCCOHT: small cell carcinoma of the ovary hypercalcemic type
NSCLC: Non-small cell lung cancer
AURKA: Aurora Kinase A
DNMT: DNA methyltransferase
EZH2: Enhancer of zest homolog 2
ABC: ATP-binding cassette
PPI: protein-protein interactions
ARM: Armadillo repeats
AU1: aryl urea-1
BRD2: Bromodomain-containing protein 2
ETS: E26 transformation-specific
IP6: Inositol hexaphosphate

## References

[B1] Abbey M. Trush V. Gibson E. Vedadi M. (2018). Targeting human retinoblastoma binding protein 4 (RBBP4) and 7 (RBBP7). bioRxiv. 10.1101/303537

[B2] Afifi N. Barrero C. A. (2023). Understanding breast cancer aggressiveness and its implications in diagnosis and treatment. MDPI 12 (4), 1375. 10.3390/jcm12041375 PMC9959372 36835911

[B3] Akimov V. Barrio-Hernandez I. Hansen S. V. F. Hallenborg P. Pedersen A. K. Bekker-Jensen D. B. (2018). UbiSite approach for comprehensive mapping of lysine and N-terminal ubiquitination sites. Nat. Struct. Mol. Biol. 25 (7), 631–640. 10.1038/s41594-018-0084-y 29967540

[B4] Allen H. F. Wade P. A. Kutateladze T. G. (2013). The NuRD architecture. Cell Mol. Life Sci. 70 (19), 3513–3524. 10.1007/s00018-012-1256-2 23340908 PMC3652912

[B5] Allo G. Bernardini M. Q. Wu R.-C. Shih I.-M. Kalloger S. Pollett A. (2014). ARID1A loss correlates with mismatch repair deficiency and intact p53 expression in high-grade endometrial carcinomas. Mod. Pathol. 27 (2), 255–261. 10.1038/modpathol.2013.144 23887303 PMC4603563

[B6] Alver B. H. Kim K. H. Lu P. Wang X. Manchester H. E. Wang W. (2017). The SWI/SNF chromatin remodelling complex is required for maintenance of lineage specific enhancers. Nat. Commun. 8 (1), 14648. 10.1038/ncomms14648 28262751 PMC5343482

[B7] Arnold M. Morgan E. Rumgay H. Mafra A. Singh D. Laversanne M. (2022). Current and future burden of breast cancer: global statistics for 2020 and 2040. Breast 66, 15–23. 10.1016/j.breast.2022.08.010 36084384 PMC9465273

[B8] Auger A. Galarneau L. Altaf M. Nourani A. Doyon Y. Utley R. T. (2008). Eaf1 is the platform for NuA4 molecular assembly that evolutionarily links chromatin acetylation to ATP-dependent exchange of histone H2A variants. Mol. Cell. Biol. 28 (7), 2257–2270. 10.1128/MCB.01755-07 18212047 PMC2268442

[B9] Auguste A. Blanc-Durand F. Deloger M. Le Formal A. Bareja R. Wilkes D. C. (2020). Small cell carcinoma of the ovary, hypercalcemic type (SCCOHT) beyond SMARCA4 mutations: a comprehensive genomic analysis. Cells 9 (6), 1496. 10.3390/cells9061496 32575483 PMC7349095

[B10] Bagchi A. Mills A. A. (2008). The quest for the 1p36 tumor suppressor. Cancer Res. 68 (8), 2551–2556. 10.1158/0008-5472.CAN-07-2095 18413720 PMC2980353

[B11] Bagchi A. Papazoglu C. Wu Y. Capurso D. Brodt M. Francis D. (2007). CHD5 is a tumor suppressor at human 1p36. Cell 128 (3), 459–475. 10.1016/j.cell.2006.11.052 17289567

[B12] Bai J. Mei P. Zhang C. Chen F. Li C. Pan Z. (2013). BRG1 is a prognostic marker and potential therapeutic target in human breast cancer. PloS One 8 (3), e59772. 10.1371/journal.pone.0059772 23533649 PMC3606107

[B13] Bao Y. Shen X. (2007). INO80 subfamily of chromatin remodeling complexes. Mutat. Res. 618 (1-2), 18–29. 10.1016/j.mrfmmm.2006.10.006 17316710 PMC2699258

[B14] Békés M. Langley D. R. Crews C. M. (2022). PROTAC targeted protein degraders: the past is prologue. Nat. Rev. Drug Discov. 21 (3), 181–200. 10.1038/s41573-021-00371-6 35042991 PMC8765495

[B15] Beltrao P. Albanèse V. Kenner L. R. Swaney D. L. Burlingame A. Villén J. (2012). Systematic functional prioritization of protein posttranslational modifications. Cell 150 (2), 413–425. 10.1016/j.cell.2012.05.036 22817900 PMC3404735

[B16] Bevill S. M. Olivares-Quintero J. F. Sciaky N. Golitz B. T. Singh D. Beltran A. S. (2019). GSK2801, a BAZ2/BRD9 bromodomain inhibitor, synergizes with BET inhibitors to induce apoptosis in triple-negative breast cancer. Mol. Cancer Res. 17 (7), 1503–1518. 10.1158/1541-7786.MCR-18-1121 31000582 PMC6610760

[B17] Bezrookove V. Khan I. A. Nosrati M. Miller J. R. McAllister S. Dar A. A. (2022). BPTF promotes the progression of distinct subtypes of breast cancer and is a therapeutic target. Front. Oncol. 12, 1011173. 10.3389/fonc.2022.1011173 36530982 PMC9748419

[B18] Bhatia N. Thareja S. (2024). Aromatase inhibitors for the treatment of breast cancer: an overview (2019–2023). Bioorg. Chem. 151, 107607. 10.1016/j.bioorg.2024.107607 39002515

[B19] Boeger H. Griesenbeck J. Strattan J. S. Kornberg R. D. (2004). Removal of promoter nucleosomes by disassembly rather than sliding *in vivo* . Mol. Cell 14 (5), 667–673. 10.1016/j.molcel.2004.05.013 15175161

[B20] Boija A. Klein I. A. Sabari B. R. Dall’Agnese A. Coffey E. L. Zamudio A. V. (2018). Transcription factors activate genes through the phase-separation capacity of their activation domains. Cell 175 (7), 1842–1855.e16. 10.1016/j.cell.2018.10.042 30449618 PMC6295254

[B21] Borges M. Linnoila R. I. Van De Velde H. J. Chen H. Nelkin B. D. Mabry M. (1997). An achaete-scute homologue essential for neuroendocrine differentiation in the lung. Nature 386 (6627), 852–855. 10.1038/386852a0 9126746

[B22] Bosse T. ter Haar N. T. Seeber L. M. v Diest P. J. Hes F. J. Vasen H. F. (2013). Loss of ARID1A expression and its relationship with PI3K-Akt pathway alterations, TP53 and microsatellite instability in endometrial cancer. Mod. Pathol. 26 (11), 1525–1535. 10.1038/modpathol.2013.96 23702729

[B23] Boyer L. A. Latek R. R. Peterson C. L. (2004). The SANT domain: a unique histone-tail-binding module? Nat. Rev. Mol. Cell Biol. 5 (2), 158–163. 10.1038/nrm1314 15040448

[B24] Brahma S. Udugama M. I. Kim J. Hada A. Bhardwaj S. K. Hailu S. G. (2017). INO80 exchanges H2A.Z for H2A by translocating on DNA proximal to histone dimers. Nat. Commun. 8 (1), 15616. 10.1038/ncomms15616 28604691 PMC5472786

[B25] Cai L. Liu B. Cao Y. Sun T. Li Y. (2023). Unveiling the molecular structure and role of RBBP4/7: implications for epigenetic regulation and cancer research. Front. Mol. Biosci. 10, 1276612. 10.3389/fmolb.2023.1276612 38028543 PMC10679446

[B26] Caldon C. E. Sergio C. M. Schütte J. Boersma M. N. Sutherland R. L. Carroll J. S. (2009). Estrogen regulation of Cyclin E2 requires Cyclin D1 but not c-Myc. Mol. Cell. Biol. 29 (17), 4623–4639. 10.1128/MCB.00269-09 19564413 PMC2725719

[B27] Cantley J. Ye X. Rousseau E. Januario T. Hamman B. D. Rose C. M. (2022). Selective PROTAC-mediated degradation of SMARCA2 is efficacious in SMARCA4 mutant cancers. Nat. Commun. 13 (1), 6814. 10.1038/s41467-022-34562-5 36357397 PMC9649729

[B28] Cao W. Liu X. Su W. Liang H. Tang H. Zhang W. (2022). LINC00665 sponges miR-641 to promote the progression of breast cancer by targeting the SNF2-related CREBBP activator protein (SRCAP). Bioengineered 13 (2), 4573–4586. 10.1080/21655979.2022.2031402 35152838 PMC8974044

[B29] Ceballos-Chávez M. Subtil-Rodríguez A. Giannopoulou E. G. Soronellas D. Vázquez-Chávez E. Vicent G. P. (2015). The chromatin remodeler CHD8 is required for activation of progesterone receptor-dependent enhancers. PLOS Genet. 11 (4), e1005174. 10.1371/journal.pgen.1005174 25894978 PMC4403880

[B30] Chabanon R. M. Morel D. Eychenne T. Colmet-Daage L. Bajrami I. Dorvault N. (2021). PBRM1 deficiency confers synthetic lethality to DNA repair inhibitors in cancer. Cancer Res. 81 (11), 2888–2902. 10.1158/0008-5472.Can-21-0628 33888468

[B31] Chen L. Cai Y. Jin J. Florens L. Swanson S. K. Washburn M. P. (2011a). Subunit organization of the human INO80 chromatin remodeling complex: an evolutionarily conserved core complex catalyzes ATP-dependent nucleosome remodeling. J. Biol. Chem. 286 (13), 11283–11289. 10.1074/jbc.M111.222505 21303910 PMC3064184

[B32] Chen Y. Chen C. Yang B. Xu Q. Wu F. Liu F. (2011b). Estrogen receptor-related genes as an important panel of predictors for breast cancer response to neoadjuvant chemotherapy. Cancer Lett. 302 (1), 63–68. 10.1016/j.canlet.2010.12.014 21220187

[B33] Chen L. Conaway R. C. Conaway J. W. (2013). Multiple modes of regulation of the human Ino80 SNF2 ATPase by subunits of the INO80 chromatin-remodeling complex. Proc. Natl. Acad. Sci. U. S. A. 110(51)**,** 20497–20502. 10.1073/pnas.1317092110 24297934 PMC3870706

[B34] Chory E. J. Kirkland J. G. Chang C.-Y. D’Andrea V. D. Gourisankar S. Dykhuizen E. C. (2020). Chemical inhibitors of a selective SWI/SNF function synergize with ATR inhibition in cancer cell killing. ACS Chem. Biol. 15 (6), 1685–1696. 10.1021/acschembio.0c00312 32369697 PMC8273930

[B35] Chu X. Guo X. Jiang Y. Yu H. Liu L. Shan W. (2017). Genotranscriptomic meta-analysis of the CHD family chromatin remodelers in human cancers - initial evidence of an oncogenic role for CHD7. Mol. Oncol. 11 (10), 1348–1360. 10.1002/1878-0261.12104 28649742 PMC5623824

[B36] Clapier C. R. (2021). Sophisticated conversations between chromatin and chromatin remodelers, and dissonances in cancer. Int. J. Mol. Sci. 22 (11), 5578. 10.3390/ijms22115578 34070411 PMC8197500

[B37] Clapier C. R. Cairns B. R. (2009). The biology of chromatin remodeling complexes. Annu. Rev. Biochem. 78, 273–304. 10.1146/annurev.biochem.77.062706.153223 19355820

[B38] Clapier C. R. Cairns B. R. (2012). Regulation of ISWI involves inhibitory modules antagonized by nucleosomal epitopes. Nature 492 (7428), 280–284. 10.1038/nature11625 23143334 PMC3631562

[B39] Clapier C. R. Iwasa J. Cairns B. R. Peterson C. L. (2017). Mechanisms of action and regulation of ATP-dependent chromatin-remodelling complexes. Nat. Rev. Mol. Cell Biol. 18 (7), 407–422. 10.1038/nrm.2017.26 28512350 PMC8127953

[B40] Cohet N. Stewart K. M. Mudhasani R. Asirvatham A. J. Mallappa C. Imbalzano K. M. (2010). SWI/SNF chromatin remodeling enzyme ATPases promote cell proliferation in normal mammary epithelial cells. J. Cell Physiol. 223 (3), 667–678. 10.1002/jcp.22072 20333683 PMC3320666

[B41] Conaway R. C. Conaway J. W. (2009). The INO80 chromatin remodeling complex in transcription, replication and repair. Trends Biochem. Sci. 34 (2), 71–77. 10.1016/j.tibs.2008.10.010 19062292

[B42] Corona D. F. Tamkun J. W. (2004). Multiple roles for ISWI in transcription, chromosome organization and DNA replication. Biochim. Biophys. Acta 1677 (1-3), 113–119. 10.1016/j.bbaexp.2003.09.018 15020052

[B43] Corona D. F. Längst G. Clapier C. R. Bonte E. J. Ferrari S. Tamkun J. W. (1999). ISWI is an ATP-dependent nucleosome remodeling factor. Mol. Cell 3 (2), 239–245. 10.1016/s1097-2765(00)80314-7 10078206

[B44] Cui Y. Bai X. Niu M. Qin Y. Zhang X. Pang D. (2019). Upregulated expression of AT-rich interactive domain-containing protein 1B predicts poor prognosis in patients with triple-negative breast cancer. Oncol. Lett. 17 (3), 3289–3295. 10.3892/ol.2019.9961 30867762 PMC6396229

[B45] Curtis C. Shah S. P. Chin S. F. Turashvili G. Rueda O. M. Dunning M. J. (2012). The genomic and transcriptomic architecture of 2,000 breast tumours reveals novel subgroups. Nature 486 (7403), 346–352. 10.1038/nature10983 22522925 PMC3440846

[B46] Cutts A. J. Soond S. M. Powell S. Chantry A. (2011). Early phase TGFβ receptor signalling dynamics stabilised by the deubiquitinase UCH37 promotes cell migratory responses. Int. J. Biochem. Cell Biol. 43 (4), 604–612. 10.1016/j.biocel.2010.12.018 21187158

[B47] D’Alesio C. Punzi S. Cicalese A. Fornasari L. Furia L. Riva L. (2016). RNAi screens identify CHD4 as an essential gene in breast cancer growth. Oncotarget 7 (49), 80901–80915. 10.18632/oncotarget.12646 27779108 PMC5348363

[B48] D'Alesio C. Bellese G. Gagliani M. C. Lechiara A. Dameri M. Grasselli E. (2019). The chromodomain helicase CHD4 regulates ERBB2 signaling pathway and autophagy in ERBB2^+^ breast cancer cells. Biol. Open 8 (4), bio038323. 10.1242/bio.038323 30967373 PMC6504000

[B49] Dahiya R. Naqvi A. Mohammad T. Alajmi M. Rehman M. Hussain A. (2019). Investigating the structural features of chromodomain proteins in the human genome and predictive impacts of their mutations in cancers. Int. J. Biol. Macromol. 131, 1101–1116. 10.1016/j.ijbiomac.2019.03.162 30917913

[B50] Dai L. Mugaanyi J. Zhang T. Tong J. Cai X. Lu C. (2022). A pan-cancer bioinformatic analysis of the carcinogenic role of SMARCA1 in human carcinomas. PLoS One 17 (9), e0274823. 10.1371/journal.pone.0274823 36126083 PMC9488775

[B51] Dang W. Bartholomew B. (2007). Domain architecture of the catalytic subunit in the ISW2-nucleosome complex. Mol. Cell Biol. 27 (23), 8306–8317. 10.1128/mcb.01351-07 17908792 PMC2169183

[B52] Dang C. V. Reddy E. P. Shokat K. M. Soucek L. (2017). Drugging the ‘undruggable’ cancer targets. Nat. Rev. Cancer 17 (8), 502–508. 10.1038/nrc.2017.36 28643779 PMC5945194

[B53] Davó-Martínez C. Helfricht A. Ribeiro-Silva C. Raams A. Tresini M. Uruci S. (2023). Different SWI/SNF complexes coordinately promote R-loop-and RAD52-dependent transcription-coupled homologous recombination. Nucleic Acids Res. 51 (17), 9055–9074. 10.1093/nar/gkad609 37470997 PMC10516656

[B54] Delmas V. Stokes D. G. Perry R. P. (1993). A mammalian DNA-binding protein that contains a chromodomain and an SNF2/SWI2-like helicase domain. Proc. Natl. Acad. Sci. U. S. A. 90(6)**,** 2414–2418. 10.1073/pnas.90.6.2414 8460153 PMC46097

[B55] Denslow S. A. Wade P. A. (2007). The human Mi-2/NuRD complex and gene regulation. Oncogene 26 (37), 5433–5438. 10.1038/sj.onc.1210611 17694084

[B56] Desmedt C. Zoppoli G. Gundem G. Pruneri G. Larsimont D. Fornili M. (2016). Genomic characterization of primary invasive lobular breast cancer. J. Clin. Oncol. 34 (16), 1872–1881. 10.1200/jco.2015.64.0334 26926684

[B57] Dhillon N. Oki M. Szyjka S. J. Aparicio O. M. Kamakaka R. T. (2006). H2A. Z functions to regulate progression through the cell cycle. Mol. Cell. Biol. 26, 489–501. 10.1128/MCB.26.2.489-501.2006 16382141 PMC1346916

[B58] Dijkwel Y. Tremethick D. J. (2022). The role of the histone variant H2A.Z in metazoan development. J. Dev. Biol. 10 (3), 28. 10.3390/jdb10030028 35893123 PMC9326617

[B59] Dinardo C. D. Fathi A. T. Kishtagari A. Bhalla K. N. Quintás-Cardama A. Reilly S. A. (2025). A phase I study of FHD-286, a dual BRG1/BRM (SMARCA4/SMARCA2) inhibitor, in patients with advanced myeloid malignancies. Clin. Cancer Res. 31 (12), 2327–2338. 10.1158/1078-0432.ccr-24-3790 40238563

[B60] Ding L. Zhao Y. Dang S. Wang Y. Li X. Yu X. (2019). Circular RNA circ-DONSON facilitates gastric cancer growth and invasion *via* NURF complex dependent activation of transcription factor SOX4. Mol. cancer 18, 45–11. 10.1186/s12943-019-1006-2 30922402 PMC6437893

[B61] DiRenzo J. Shang Y. Phelan M. Sif S. Myers M. Kingston R. (2000). BRG-1 is recruited to estrogen-responsive promoters and cooperates with factors involved in histone acetylation. Mol. Cell Biol. 20 (20), 7541–7549. 10.1128/mcb.20.20.7541-7549.2000 11003650 PMC86306

[B62] Doroshow D. B. Eder J. P. LoRusso P. M. (2017). BET inhibitors: a novel epigenetic approach. Ann. Oncol. 28 (8), 1776–1787. 10.1093/annonc/mdx157 28838216

[B63] Dreier M. R. Walia J. de la Serna I. L. (2024). Targeting SWI/SNF complexes in cancer: pharmacological approaches and implications. Epigenomes 8 (1), 7. 10.3390/epigenomes8010007 38390898 PMC10885108

[B64] Dubey R. Lebensohn A. M. Bahrami-Nejad Z. Marceau C. Champion M. Gevaert O. (2016). Chromatin-remodeling complex SWI/SNF controls multidrug resistance by transcriptionally regulating the drug efflux pump ABCB1. Cancer Res. 76 (19), 5810–5821. 10.1158/0008-5472.CAN-16-0716 27503929 PMC5050136

[B65] Duplaquet L. So K. Ying A. W. Pal Choudhuri S. Li X. Xu G. D. (2024). Mammalian SWI/SNF complex activity regulates POU2F3 and constitutes a targetable dependency in small cell lung cancer. Cancer Cell 42 (8), 1352–1369.e13. 10.1016/j.ccell.2024.06.012 39029464 PMC11494612

[B66] Dutta P. Tanti G. K. Sharma S. Goswami S. K. Komath S. S. Mayo M. W. (2012). Global epigenetic changes induced by SWI2/SNF2 inhibitors characterize neomycin-resistant mammalian cells. PloS One 7 (11), e49822. 10.1371/journal.pone.0049822 23209606 PMC3509132

[B67] D’Alesio C. Bellese G. Gagliani M. C. Aiello C. Grasselli E. Marcocci G. (2017). Cooperative antitumor activities of carnosic acid and trastuzumab in ERBB2+ breast cancer cells. J. Exp. Clin. Cancer Res. 36, 154–16. 10.1186/s13046-017-0615-0 29100552 PMC5670707

[B68] Erdel F. Schubert T. Marth C. Längst G. Rippe K. (2010). Human ISWI chromatin-remodeling complexes sample nucleosomes *via* transient binding reactions and become immobilized at active sites. Proc. Natl. Acad. Sci. U. S. A. 107(46)**,** 19873–19878. 10.1073/pnas.1003438107 20974961 PMC2993390

[B69] Fang F. M. Li C. F. Huang H. Y. Lai M. T. Chen C. M. Chiu I. W. (2011). Overexpression of a chromatin remodeling factor, RSF-1/HBXAP, correlates with aggressive oral squamous cell carcinoma. Am. J. Pathol. 178 (5), 2407–2415. 10.1016/j.ajpath.2011.01.043 21514451 PMC3081206

[B70] Farnaby W. Koegl M. Roy M. J. Whitworth C. Diers E. Trainor N. (2019). BAF complex vulnerabilities in cancer demonstrated *via* structure-based PROTAC design. Nat. Chem. Biol. 15 (7), 672–680. 10.1038/s41589-019-0294-6 31178587 PMC6600871

[B71] Fei J. Torigoe S. E. Brown C. R. Khuong M. T. Kassavetis G. A. Boeger H. (2015). The prenucleosome, a stable conformational isomer of the nucleosome. Genes Dev. 29 (24), 2563–2575. 10.1101/gad.272633.115 26680301 PMC4699385

[B72] Feng Y. Tian Y. Wu Z. Xu Y. (2018). Cryo-EM structure of human SRCAP complex. Cell Res. 28 (11), 1121–1123. 10.1038/s41422-018-0102-y 30337683 PMC6218446

[B73] Ferlay J. E. M. Lam F. Laversanne M. Colombet M. Mery L. Piñeros M. (2024). Global cancer observatory: cancer today *.* Lyon, France: International Agency for Research on Cancer. Available online at: https://gco.iarc.who.int/today, (Accessed 11 July 2024).

[B74] Filippakopoulos P. Picaud S. Mangos M. Keates T. Lambert J.-P. Barsyte-Lovejoy D. (2012). Histone recognition and large-scale structural analysis of the human bromodomain family. Cell 149 (1), 214–231. 10.1016/j.cell.2012.02.013 22464331 PMC3326523

[B75] Flaus A. Martin D. M. Barton G. J. Owen-Hughes T. (2006). Identification of multiple distinct Snf2 subfamilies with conserved structural motifs. Nucleic Acids Res. 34 (10), 2887–2905. 10.1093/nar/gkl295 16738128 PMC1474054

[B76] Fontana B. Gallerani G. Salamon I. Pace I. Roncarati R. Ferracin M. (2023). ARID1A in cancer: friend or foe? Front. Oncol. 13, 1136248. 10.3389/fonc.2023.1136248 36890819 PMC9987588

[B77] Frey W. D. Chaudhry A. Slepicka P. F. Ouellette A. M. Kirberger S. E. Pomerantz W. C. (2017). BPTF maintains chromatin accessibility and the self-renewal capacity of mammary gland stem cells. Stem Cell Rep. 9 (1), 23–31. 10.1016/j.stemcr.2017.04.031 28579392 PMC5783326

[B78] Fukumoto T. Park P. H. Wu S. Fatkhutdinov N. Karakashev S. Nacarelli T. (2018). Repurposing pan-HDAC inhibitors for ARID1A-mutated ovarian cancer. Cell Rep. 22 (13), 3393–3400. 10.1016/j.celrep.2018.03.019 29590609 PMC5903572

[B79] Fukumoto T. Fatkhutdinov N. Zundell J. A. Tcyganov E. N. Nacarelli T. Karakashev S. (2019). HDAC6 inhibition synergizes with anti-PD-L1 therapy in ARID1A-inactivated ovarian cancer. Cancer Res. 79 (21), 5482–5489. 10.1158/0008-5472.CAN-19-1302 31311810 PMC6825538

[B80] García-Pedrero J. M. Kiskinis E. Parker M. G. Belandia B. (2006). The SWI/SNF chromatin remodeling subunit BAF57 is a critical regulator of estrogen receptor function in breast cancer cells. J. Biol. Chem. 281 (32), 22656–22664. 10.1074/jbc.m602561200 16769725

[B81] Gatchalian J. Malik S. Ho J. Lee D. S. Kelso T. W. R. Shokhirev M. N. (2018). A non-canonical BRD9-containing BAF chromatin remodeling complex regulates naive pluripotency in mouse embryonic stem cells. Nat. Commun. 9 (1), 5139. 10.1038/s41467-018-07528-9 30510198 PMC6277444

[B82] Ghodsi R. Hemmateenejad B. (2016). QSAR study of diarylalkylimidazole and diarylalkyltriazole aromatase inhibitors. Med. Chem. Res. 25 (5), 834–842. 10.1007/s00044-016-1530-1

[B83] Glancy E. Ciferri C. Bracken A. P. (2021). Structural basis for PRC2 engagement with chromatin. Curr. Opin. Struct. Biol. 67, 135–144. 10.1016/j.sbi.2020.10.017 33232890

[B84] Grüne T. Brzeski J. Eberharter A. Clapier C. R. Corona D. F. Becker P. B. (2003). Crystal structure and functional analysis of a nucleosome recognition module of the remodeling factor ISWI. Mol. Cell 12 (2), 449–460. 10.1016/s1097-2765(03)00273-9 14536084

[B85] Guo X. Zhang Y. Mayakonda A. Madan V. Ding L.-W. Lin L.-H. (2018). ARID1A and CEBPα cooperatively inhibit UCA1 transcription in breast cancer. Oncogene 37 (45), 5939–5951. 10.1038/s41388-018-0371-4 29980791

[B86] Guo A. Huang H. Zhu Z. Chen M. J. Shi H. Yuan S. (2022). cBAF complex components and MYC cooperate early in CD8^+^ T cell fate. Nature 607 (7917), 135–141. 10.1038/s41586-022-04849-0 35732731 PMC9623036

[B87] Gurard-Levin Z. A. Quivy J. P. Almouzni G. (2014). Histone chaperones: assisting histone traffic and nucleosome dynamics. Annu. Rev. Biochem. 83, 487–517. 10.1146/annurev-biochem-060713-035536 24905786

[B88] Hagiwara M. Fushimi A. Yamashita N. Bhattacharya A. Rajabi H. Long M. D. (2021). MUC1-C activates the PBAF chromatin remodeling complex in integrating redox balance with progression of human prostate cancer stem cells. Oncogene 40 (30), 4930–4940. 10.1038/s41388-021-01899-y 34163028 PMC8321896

[B89] Hauk G. McKnight J. N. Nodelman I. M. Bowman G. D. (2010). The chromodomains of the Chd1 chromatin remodeler regulate DNA access to the ATPase motor. Mol. Cell 39 (5), 711–723. 10.1016/j.molcel.2010.08.012 20832723 PMC2950701

[B90] He T. Xiao L. Qiao Y. Klingbeil O. Young E. Wu X. S. (2024). Targeting the mSWI/SNF complex in POU2F-POU2AF transcription factor-driven malignancies. Cancer Cell 42 (8), 1336–1351.e9. 10.1016/j.ccell.2024.06.006 39029462 PMC12147762

[B91] Helming K. C. Wang X. Wilson B. G. Vazquez F. Haswell J. R. Manchester H. E. (2014). ARID1B is a specific vulnerability in ARID1A-mutant cancers. Nat. Med. 20 (3), 251–254. 10.1038/nm.3480 24562383 PMC3954704

[B92] Ho L. Crabtree G. R. (2010). Chromatin remodelling during development. Nature 463 (7280), 474–484. 10.1038/nature08911 20110991 PMC3060774

[B93] Hochheimer A. Zhou S. Zheng S. Holmes M. C. Tjian R. (2002). TRF2 associates with DREF and directs promoter-selective gene expression in *Drosophila* . Nature 420 (6914), 439–445. 10.1038/nature01167 12459787

[B94] Hoffman G. R. Rahal R. Buxton F. Xiang K. McAllister G. Frias E. (2014). Functional epigenetics approach identifies BRM/SMARCA2 as a critical synthetic lethal target in BRG1-deficient cancers. Proc. Natl. Acad. Sci. U. S. A. 111 (8), 3128–3133. 10.1073/pnas.1316793111 24520176 PMC3939885

[B95] Hopson S. Thompson M. J. (2017). BAF180: its roles in DNA repair and consequences in cancer. ACS Chem. Biol. 12 (10), 2482–2490. 10.1021/acschembio.7b00541 28921948

[B96] Hou M.-F. Luo C.-W. Chang T.-M. Hung W.-C. Chen T.-Y. Tsai Y.-L. (2017). The NuRD complex-mediated p21 suppression facilitates chemoresistance in BRCA-proficient breast cancer. Exp. Cell Res. 359 (2), 458–465. 10.1016/j.yexcr.2017.08.029 28842166

[B97] Hu K. Wu W. Li Y. Lin L. Chen D. Yan H. (2019). Poly (ADP‐ribosyl) ation of BRD 7 by PARP 1 confers resistance to DNA‐damaging chemotherapeutic agents. EMBO Rep. 20 (5), e46166. 10.15252/embr.201846166 30940648 PMC6500972

[B98] Huang Y.-H. Klingbeil O. He X.-Y. Wu X. S. Arun G. Lu B. (2018). POU2F3 is a master regulator of a tuft cell-like variant of small cell lung cancer. Genes Dev. 32 (13-14), 915–928. 10.1101/gad.314815.118 29945888 PMC6075037

[B99] Hurtado A. Holmes K. A. Ross-Innes C. S. Schmidt D. Carroll J. S. (2011). FOXA1 is a key determinant of estrogen receptor function and endocrine response. Nat. Genet. 43 (1), 27–33. 10.1038/ng.730 21151129 PMC3024537

[B100] Ito T. Bulger M. Pazin M. J. Kobayashi R. Kadonaga J. T. (1997). ACF, an ISWI-containing and ATP-utilizing chromatin assembly and remodeling factor. Cell 90 (1), 145–155. 10.1016/s0092-8674(00)80321-9 9230310

[B101] Ito T. Levenstein M. E. Fyodorov D. V. Kutach A. K. Kobayashi R. Kadonaga J. T. (1999). ACF consists of two subunits, Acf1 and ISWI, that function cooperatively in the ATP-dependent catalysis of chromatin assembly. Genes Dev. 13 (12), 1529–1539. 10.1101/gad.13.12.1529 10385622 PMC316812

[B102] Jacquet K. Fradet-Turcotte A. Avvakumov N. Lambert J.-P. Roques C. Pandita R. K. (2016). The TIP60 complex regulates bivalent chromatin recognition by 53BP1 through direct H4K20me binding and H2AK15 acetylation. Mol. Cell 62 (3), 409–421. 10.1016/j.molcel.2016.03.031 27153538 PMC4887106

[B103] Jin J. Cai Y. Yao T. Gottschalk A. J. Florens L. Swanson S. K. (2005). A mammalian chromatin remodeling complex with similarities to the yeast INO80 complex. J. Biol. Chem. 280 (50), 41207–41212. 10.1074/jbc.M509128200 16230350

[B104] Jin Q. Mao X. Li B. Guan S. Yao F. Jin F. (2015). Overexpression of SMARCA5 correlates with cell proliferation and migration in breast cancer. Tumour Biol. 36 (3), 1895–1902. 10.1007/s13277-014-2791-2 25377162

[B105] Jones C. A. Tansey W. P. Weissmiller A. M. (2022). Emerging themes in mechanisms of tumorigenesis by SWI/SNF subunit mutation. Epigenet Insights 15, 25168657221115656. 10.1177/25168657221115656 35911061 PMC9329810

[B106] Kabeya Y. Mizushima N. Ueno T. Yamamoto A. Kirisako T. Noda T. (2000). LC3, a mammalian homologue of yeast Apg8p, is localized in autophagosome membranes after processing. Embo J. 19 (21), 5720–5728. 10.1093/emboj/19.21.5720 11060023 PMC305793

[B107] Kabeya Y. Mizushima N. Yamamoto A. Oshitani-Okamoto S. Ohsumi Y. Yoshimori T. (2004). LC3, GABARAP and GATE16 localize to autophagosomal membrane depending on form-II formation. J. Cell Sci. 117 (Pt 13), 2805–2812. 10.1242/jcs.01131 15169837

[B108] Kadoch C. Crabtree G. R. (2015). Mammalian SWI/SNF chromatin remodeling complexes and cancer: mechanistic insights gained from human genomics. Sci. Adv. 1(5)**,** e1500447. 10.1126/sciadv.1500447 26601204 PMC4640607

[B109] Kadoch C. Hargreaves D. C. Hodges C. Elias L. Ho L. Ranish J. (2013). Proteomic and bioinformatic analysis of mammalian SWI/SNF complexes identifies extensive roles in human malignancy. Nat. Genet. 45 (6), 592–601. 10.1038/ng.2628 23644491 PMC3667980

[B110] Kaelin W. G. (2009). Synthetic lethality: a framework for the development of wiser cancer therapeutics. Genome Med. 1, 99–6. 10.1186/gm99 19863774 PMC2784312

[B111] Kasten M. M. Clapier C. R. Cairns B. R. (2011). SnapShot: chromatin remodeling: SWI/SNF. Cell 144 (2), 310.e1. 10.1016/j.cell.2011.01.007 21241897

[B112] Kim J. H. Kim B. Cai L. Choi H. J. Ohgi K. A. Tran C. (2005). Transcriptional regulation of a metastasis suppressor gene by Tip60 and β-catenin complexes. Nature 434 (7035), 921–926. 10.1038/nature03452 15829968

[B113] Kobor M. S. Venkatasubrahmanyam S. Meneghini M. D. Gin J. W. Jennings J. L. Link A. J. (2004). A protein complex containing the conserved Swi2/Snf2-related ATPase Swr1p deposits histone variant H2A. Z into euchromatin. PLoS Biol. 2 (5), e131. 10.1371/journal.pbio.0020131 15045029 PMC374244

[B114] Koch J. G. Gu X. Han Y. El-Naggar A. K. Olson M. V. Medina D. (2007). Mammary tumor modifiers in BALB/cJ mice heterozygous for p53. Mamm. Genome 18, 300–309. 10.1007/s00335-007-9028-2 17557176

[B115] Koedoot E. Fokkelman M. Rogkoti V. M. Smid M. van de Sandt I. de Bont H. (2019). Uncovering the signaling landscape controlling breast cancer cell migration identifies novel metastasis driver genes. Nat. Commun. 10 (1), 2983. 10.1038/s41467-019-11020-3 31278301 PMC6611796

[B116] Kolla V. Naraparaju K. Zhuang T. Higashi M. Kolla S. Blobel G. A. (2015). The tumour suppressor CHD5 forms a NuRD-type chromatin remodelling complex. Biochem. J. 468 (2), 345–352. 10.1042/BJ20150030 25825869 PMC4487910

[B117] Konev A. Y. Tribus M. Park S. Y. Podhraski V. Lim C. Y. Emelyanov A. V. (2007). CHD1 motor protein is required for deposition of histone variant H3.3 into chromatin *in vivo* . Science 317 (5841), 1087–1090. 10.1126/science.1145339 17717186 PMC3014568

[B118] Konrat R. (2014). NMR contributions to structural dynamics studies of intrinsically disordered proteins. J. Magnetic Reson. 241, 74–85. 10.1016/j.jmr.2013.11.011 24656082 PMC3985426

[B119] Kotagiri S. Wang Y. Han Y. Liang X. Blazanin N. Mazhar H. (2025). Discovery of novel, potent, and orally bioavailable SMARCA2 proteolysis-targeting chimeras with synergistic antitumor activity in combination with Kirsten Rat sarcoma viral oncogene homologue G12C inhibitors. J. Med. Chem. 68 (9), 9202–9219. 10.1021/acs.jmedchem.4c02577 40280558 PMC12067438

[B120] Krogan N. J. Keogh M.-C. Datta N. Sawa C. Ryan O. W. Ding H. (2003). A Snf2 family ATPase complex required for recruitment of the histone H2A variant Htz1. Mol. Cell 12 (6), 1565–1576. 10.1016/s1097-2765(03)00497-0 14690608

[B121] Kshirsagar M. Jiang W. Shih Ie M. (2012). DNA damage response is prominent in ovarian high-grade serous carcinomas, especially those with Rsf-1 (HBXAP) overexpression. J. Oncol. 2012, 621685. 10.1155/2012/621685 22028712 PMC3199114

[B122] Kukimoto I. Elderkin S. Grimaldi M. Oelgeschläger T. Varga-Weisz P. D. (2004). The histone-fold protein complex CHRAC-15/17 enhances nucleosome sliding and assembly mediated by ACF. Mol. Cell 13 (2), 265–277. 10.1016/s1097-2765(03)00523-9 14759371

[B123] Kumar R. Li D.-Q. Müller S. Knapp S. (2016). Epigenomic regulation of oncogenesis by chromatin remodeling. Oncogene 35 (34), 4423–4436. 10.1038/onc.2015.513 26804164

[B124] Kunert N. Brehm A. (2009). Novel Mi-2 related ATP-dependent chromatin remodelers. Epigenetics 4 (4), 209–211. 10.4161/epi.8933 19535903

[B125] Lan L. Ui A. Nakajima S. Hatakeyama K. Hoshi M. Watanabe R. (2010). The ACF1 complex is required for DNA double-strand break repair in human cells. Mol. Cell 40 (6), 976–987. 10.1016/j.molcel.2010.12.003 21172662

[B126] Landry J. W. Banerjee S. Taylor B. Aplan P. D. Singer A. Wu C. (2011). Chromatin remodeling complex NURF regulates thymocyte maturation. Genes Dev. 25 (3), 275–286. 10.1101/gad.2007311 21289071 PMC3034902

[B127] Lee D. Lee D. Y. Hwang Y. S. Seo H. R. Lee S. A. Kwon J. (2021). The bromodomain inhibitor PFI-3 sensitizes cancer cells to DNA damage by targeting SWI/SNF. Mol. Cancer Res. 19 (5), 900–912. 10.1158/1541-7786.Mcr-20-0289 33208498

[B128] Lee R. S. Sad K. Fawwal D. V. Spangle J. M. (2023). Emerging role of epigenetic modifiers in breast cancer pathogenesis and therapeutic response. Cancers (Basel) 15 (15), 4005. 10.3390/cancers15154005 37568822 PMC10417282

[B129] Li J. J. Lee C. S. (2023). The role of the AT-Rich interaction domain 1A gene (ARID1A) in human carcinogenesis. Genes (Basel) 15 (1), 5. 10.3390/genes15010005 38275587 PMC10815128

[B130] Li M. Aliotta J. M. Asara J. M. Wu Q. Dooner M. S. Tucker L. D. (2010). Intercellular transfer of proteins as identified by stable isotope labeling of amino acids in cell culture. J. Biol. Chem. 285 (9), 6285–6297. 10.1074/jbc.M109.057943 20026604 PMC2825424

[B131] Li H. J. Yu P. N. Huang K. Y. Su H. Y. Hsiao T. H. Chang C. P. (2016). NKX6.1 functions as a metastatic suppressor through epigenetic regulation of the epithelial-mesenchymal transition. Oncogene 35 (17), 2266–2278. 10.1038/onc.2015.289 26257059 PMC4855079

[B132] Li X. Ding D. Yao J. Zhou B. Shen T. Qi Y. (2019). Chromatin remodeling factor BAZ1A regulates cellular senescence in both cancer and normal cells. Life Sci. 229, 225–232. 10.1016/j.lfs.2019.05.023 31085244

[B133] Li Y. Gong H. Wang P. Zhu Y. Peng H. Cui Y. (2021). The emerging role of ISWI chromatin remodeling complexes in cancer. J. Exp. Clin. Cancer Res. 40 (1), 346. 10.1186/s13046-021-02151-x 34736517 PMC8567610

[B134] Li K. Wang B. Hu H. (2024a). Research progress of SWI/SNF complex in breast cancer. Epigenetics Chromatin 17 (1), 4. 10.1186/s13072-024-00531-z 38365747 PMC10873968

[B135] Li S. Wang L. Shi J. Chen Y. Xiao A. Huo B. (2024b). Chromatin accessibility complex subunit 1 enhances tumor growth by regulating the oncogenic transcription of YAP in breast and cervical cancer. PeerJ 12, e16752. 10.7717/peerj.16752 38223760 PMC10787542

[B136] Liang X. Cao Y. Duan Z. Wang M. Zhang N. Ding Y. (2023). Discovery of new small molecule inhibitors of the BPTF bromodomain. Bioorg. Chem. 134, 106453. 10.1016/j.bioorg.2023.106453 36898211

[B137] Lippai M. Lőw P. (2014). The role of the selective adaptor p62 and ubiquitin-like proteins in autophagy. Biomed. Res. Int. 2014, 832704. 10.1155/2014/832704 25013806 PMC4075091

[B138] Liu W. J. Ye L. Huang W. F. Guo L. J. Xu Z. G. Wu H. L. (2016). p62 links the autophagy pathway and the ubiqutin–proteasome system upon ubiquitinated protein degradation. Cell. Mol. Biol. Lett. 21 (1), 29. 10.1186/s11658-016-0031-z 28536631 PMC5415757

[B139] Low J. K. Webb S. R. Silva A. P. Saathoff H. Ryan D. P. Torrado M. (2016). CHD4 is a peripheral component of the nucleosome remodeling and deacetylase complex. J. Biol. Chem. 291 (30), 15853–15866. 10.1074/jbc.M115.707018 27235397 PMC4957066

[B140] Lundqvist J. Hansen S. K. Lykkesfeldt A. E. (2013). Vitamin D analog EB1089 inhibits aromatase expression by dissociation of comodulator WSTF from the CYP19A1 promoter-a new regulatory pathway for aromatase. Biochim. Biophys. Acta 1833 (1), 40–47. 10.1016/j.bbamcr.2012.10.012 23085504

[B141] Lundqvist J. Kirkegaard T. Laenkholm A. V. Duun-Henriksen A. K. Bak M. Feldman D. (2018). Williams syndrome transcription factor (WSTF) acts as an activator of estrogen receptor signaling in breast cancer cells and the effect can be abrogated by 1α,25-dihydroxyvitamin D(3). J. Steroid Biochem. Mol. Biol. 177, 171–178. 10.1016/j.jsbmb.2017.06.003 28610873

[B142] Luo C.-W. Wu C.-C. Chang S.-J. Chang T.-M. Chen T.-Y. Chai C.-Y. (2018). CHD4-mediated loss of E-cadherin determines metastatic ability in triple-negative breast cancer cells. Exp. Cell Res. 363 (1), 65–72. 10.1016/j.yexcr.2017.12.032 29305962

[B143] Lusser A. Urwin D. L. Kadonaga J. T. (2005). Distinct activities of CHD1 and ACF in ATP-dependent chromatin assembly. Nat. Struct. Mol. Biol. 12 (2), 160–166. 10.1038/nsmb884 15643425

[B144] Mahanic C. S. Budhavarapu V. Graves J. D. Li G. Lin W.-C. (2015). Regulation of E2 promoter binding factor 1 (E2F1) transcriptional activity through a deubiquitinating enzyme, UCH37. J. Biol. Chem. 290 (44), 26508–26522. 10.1074/jbc.M115.659425 26396186 PMC4646310

[B145] Mahmood S. F. Gruel N. Chapeaublanc E. Lescure A. Jones T. Reyal F. (2014). A siRNA screen identifies RAD21, EIF3H, CHRAC1 and TANC2 as driver genes within the 8q23, 8q24.3 and 17q23 amplicons in breast cancer with effects on cell growth, survival and transformation. Carcinogenesis 35 (3), 670–682. 10.1093/carcin/bgt351 24148822

[B146] Mani U. S A. S. Goutham R. N. A. Mohan S. S. (2017). SWI/SNF Infobase-An exclusive information portal for SWI/SNF remodeling complex subunits. PLoS One 12 (9), e0184445. 10.1371/journal.pone.0184445 28961249 PMC5621669

[B147] Marfella C. G. A. Imbalzano A. N. (2007). The Chd family of chromatin remodelers. Mutat. Research/Fundamental Mol. Mech. Mutagen. 618 (1-2), 30–40. 10.1016/j.mrfmmm.2006.07.012 17350655 PMC1899158

[B148] Marfella C. G. Ohkawa Y. Coles A. H. Garlick D. S. Jones S. N. Imbalzano A. N. (2006). Mutation of the SNF2 family member Chd2 affects mouse development and survival. J. Cell. Physiol. 209 (1), 162–171. 10.1002/jcp.20718 16810678

[B149] Martin B. J. E. Ablondi E. F. Goglia C. Mimoso C. A. Espinel-Cabrera P. R. Adelman K. (2023). Global identification of SWI/SNF targets reveals compensation by EP400. Cell 186 (24), 5290–5307.e26. 10.1016/j.cell.2023.10.006 37922899 PMC11307202

[B150] Mathur R. Alver B. H. San Roman A. K. Wilson B. G. Wang X. Agoston A. T. (2017). ARID1A loss impairs enhancer-mediated gene regulation and drives colon cancer in mice. Nat. Genet. 49 (2), 296–302. 10.1038/ng.3744 27941798 PMC5285448

[B151] Mayes K. Qiu Z. Alhazmi A. Landry J. W. (2014). ATP-dependent chromatin remodeling complexes as novel targets for cancer therapy. Adv. Cancer Res. 121, 183–233. 10.1016/b978-0-12-800249-0.00005-6 24889532 PMC4839282

[B152] Mayes K. Alkhatib S. G. Peterson K. Alhazmi A. Song C. Chan V. (2016). BPTF depletion enhances T-cell-mediated antitumor immunity. Cancer Res. 76 (21), 6183–6192. 10.1158/0008-5472.Can-15-3125 27651309 PMC5093041

[B153] Mayes K. Elsayed Z. Alhazmi A. Waters M. Alkhatib S. G. Roberts M. (2017). BPTF inhibits NK cell activity and the abundance of natural cytotoxicity receptor co-ligands. Oncotarget 8 (38), 64344–64357. 10.18632/oncotarget.17834 28969075 PMC5610007

[B154] Miao D. Margolis C. A. Gao W. Voss M. H. Li W. Martini D. J. (2018). Genomic correlates of response to immune checkpoint therapies in clear cell renal cell carcinoma. Science 359 (6377), 801–806. 10.1126/science.aan5951 29301960 PMC6035749

[B155] Michel B. C. D'Avino A. R. Cassel S. H. Mashtalir N. McKenzie Z. M. McBride M. J. (2018). A non-canonical SWI/SNF complex is a synthetic lethal target in cancers driven by BAF complex perturbation. Nat. Cell Biol. 20 (12), 1410–1420. 10.1038/s41556-018-0221-1 30397315 PMC6698386

[B156] Mills A. A. (2017). The chromodomain helicase DNA-binding chromatin remodelers: family traits that protect from and promote cancer. Cold Spring Harb. Perspect. Med. 7 (4), a026450. 10.1101/cshperspect.a026450 28096241 PMC5378010

[B157] Min J.-N. Tian Y. Xiao Y. Wu L. Li L. Chang S. (2013). The mINO80 chromatin remodeling complex is required for efficient telomere replication and maintenance of genome stability. Cell Res. 23 (12), 1396–1413. 10.1038/cr.2013.113 23979016 PMC3847565

[B158] Mittal P. Roberts C. W. M. (2020). The SWI/SNF complex in cancer - biology, biomarkers and therapy. Nat. Rev. Clin. Oncol. 17 (7), 435–448. 10.1038/s41571-020-0357-3 32303701 PMC8723792

[B159] Mizuguchi G. Shen X. Landry J. Wu W. H. Sen S. Wu C. (2004). ATP-driven exchange of histone H2AZ variant catalyzed by SWR1 chromatin remodeling complex. Science 303 (5656), 343–348. 10.1126/science.1090701 14645854

[B160] Mo D. Li C. Liang J. Shi Q. Su N. Luo S. (2015). Low PBRM1 identifies tumor progression and poor prognosis in breast cancer. Int. J. Clin. Exp. Pathol. 8 (8), 9307–9313. Available online at: https://pmc.ncbi.nlm.nih.gov/articles/PMC4583913/. 26464681 PMC4583913

[B161] Mohrmann L. Verrijzer C. P. (2005a). Composition and functional specificity of SWI2/SNF2 class chromatin remodeling complexes. Biochim. Biophys. Acta 1681, 59–73. 10.1016/j.bbaexp.2004.10.005 15627498

[B162] Moody R. R. Lo M. C. Meagher J. L. Lin C. C. Stevers N. O. Tinsley S. L. (2018). Probing the interaction between the histone methyltransferase/deacetylase subunit RBBP4/7 and the transcription factor BCL11A in epigenetic complexes. J. Biol. Chem. 293 (6), 2125–2136. 10.1074/jbc.M117.811463 29263092 PMC5808772

[B163] Mulero-Navarro S. Esteller M. (2008). Chromatin remodeling factor CHD5 is silenced by promoter CpG island hypermethylation in human cancer. Epigenetics 3 (4), 210–215. 10.4161/epi.3.4.6610 18698156

[B164] Murawska M. Brehm A. (2011). CHD chromatin remodelers and the transcription cycle. Transcription 2 (6), 244–253. 10.4161/trns.2.6.17840 22223048 PMC3265784

[B165] Muthuswami R. Mesner L. D. Wang D. Hill D. A. Imbalzano A. N. Hockensmith J. W. (2000). Phosphoaminoglycosides inhibit SWI2/SNF2 family DNA-dependent molecular motor domains. Biochemistry 39 (15), 4358–4365. 10.1021/bi992503r 10757984

[B166] Nagarajan S. Rao S. V. Sutton J. Cheeseman D. Dunn S. Papachristou E. K. (2020). ARID1A influences HDAC1/BRD4 activity, intrinsic proliferative capacity and breast cancer treatment response. Nat. Genet. 52 (2), 187–197. 10.1038/s41588-019-0541-5 31913353 PMC7116647

[B167] Nagl N. G. Jr. Wang X. Patsialou A. Van Scoy M. Moran E. (2007). Distinct mammalian SWI/SNF chromatin remodeling complexes with opposing roles in cell-cycle control. Embo J. 26 (3), 752–763. 10.1038/sj.emboj.7601541 17255939 PMC1794396

[B168] Nakayama R. T. Pulice J. L. Valencia A. M. McBride M. J. McKenzie Z. M. Gillespie M. A. (2017). SMARCB1 is required for widespread BAF complex-mediated activation of enhancers and bivalent promoters. Nat. Genet. 49 (11), 1613–1623. 10.1038/ng.3958 28945250 PMC5803080

[B169] Narlikar G. J. Fan H.-Y. Kingston R. E. (2002). Cooperation between complexes that regulate chromatin structure and transcription. Cell 108 (4), 475–487. 10.1016/S0092-8674(02)00654-2 11909519

[B170] Narlikar G. J. Sundaramoorthy R. Owen-Hughes T. (2013). Mechanisms and functions of ATP-dependent chromatin-remodeling enzymes. Cell 154 (3), 490–503. 10.1016/j.cell.2013.07.011 23911317 PMC3781322

[B171] Navin N. Krasnitz A. Rodgers L. Cook K. Meth J. Kendall J. (2010). Inferring tumor progression from genomic heterogeneity. Genome Res. 20 (1), 68–80. 10.1101/gr.099622.109 19903760 PMC2798832

[B172] Nijman S. M. (2011). Synthetic lethality: general principles, utility and detection using genetic screens in human cells. FEBS Lett. 585 (1), 1–6. 10.1016/j.febslet.2010.11.024 21094158 PMC3018572

[B173] Nio K. Yamashita T. Okada H. Kondo M. Hayashi T. Hara Y. (2015). Defeating EpCAM^+^ liver cancer stem cells by targeting chromatin remodeling enzyme CHD4 in human hepatocellular carcinoma. J. Hepatol. 63 (5), 1164–1172. 10.1016/j.jhep.2015.06.009 26095183

[B310] Oppikofer M. Bai T. Gan Y. Haley B. Liu B. Sandoval W. (2017). Expansion of the ISWI chromatin remodeler family with new active complexes. EMBO Rep. 18 (10), 1697–1706. 10.15252/embr.201744011 28801535 PMC5623870

[B174] Ordonez-Rubiano S. C. Strohmier B. P. Sood S. Dykhuizen E. C. (2024). SWI/SNF chromatin remodelers in prostate cancer progression. Front. Epigenetics Epigenomics 1, 1337345. 10.3389/freae.2023.1337345

[B175] Ordureau A. Paulo J. A. Zhang J. An H. Swatek K. N. Cannon J. R. (2020). Global landscape and dynamics of Parkin and USP30-Dependent ubiquitylomes in iNeurons during mitophagic signaling. Mol. Cell 77 (5), 1124–1142.e10. 10.1016/j.molcel.2019.11.013 32142685 PMC7098486

[B176] Ou-Yang F. Pan M.-R. Chang S.-J. Wu C.-C. Fang S.-Y. Li C.-L. (2019). Identification of CHD4-β1 integrin axis as a prognostic marker in triple-negative breast cancer using next-generation sequencing and bioinformatics. Life Sci. 238, 116963. 10.1016/j.lfs.2019.116963 31639396

[B177] O’Shaughnessy A. Hendrich B. (2013). CHD4 in the DNA-damage response and cell cycle progression: not so NuRDy now. Biochem. Soc. Trans. 41 (3), 777–782. 10.1042/BST20130027 23697937 PMC3685327

[B178] Pan D. Kobayashi A. Jiang P. Ferrari de Andrade L. Tay R. E. Luoma A. M. (2018). A major chromatin regulator determines resistance of tumor cells to T cell-mediated killing. Science 359 (6377), 770–775. 10.1126/science.aao1710 29301958 PMC5953516

[B179] Pan J. McKenzie Z. M. D’Avino A. R. Mashtalir N. Lareau C. A. St. Pierre R. (2019). The ATPase module of mammalian SWI/SNF family complexes mediates subcomplex identity and catalytic activity–independent genomic targeting. Nat. Genet. 51 (4), 618–626. 10.1038/s41588-019-0363-5 30858614 PMC6755913

[B180] Pancione M. Remo A. Zanella C. Sabatino L. Blasi A. D. Laudanna C. (2013). The chromatin remodelling component SMARCB1/INI1 influences the metastatic behavior of colorectal cancer through a gene signature mapping to chromosome 22. J. Transl. Med. 11 (1), 297. 10.1186/1479-5876-11-297 24286138 PMC4220786

[B181] Papamichos-Chronakis M. Watanabe S. Rando O. J. Peterson C. L. (2011). Global regulation of H2A. Z localization by the INO80 chromatin-remodeling enzyme is essential for genome integrity. Cell 144 (2), 200–213. 10.1016/j.cell.2010.12.021 21241891 PMC3035940

[B182] Papillon J. P. Nakajima K. Adair C. D. Hempel J. Jouk A. O. Karki R. G. (2018). Discovery of orally active inhibitors of brahma homolog (BRM)/SMARCA2 ATPase activity for the treatment of brahma related gene 1 (BRG1)/SMARCA4-mutant cancers. J. Med. Chem. 61 (22), 10155–10172. 10.1021/acs.jmedchem.8b01318 30339381

[B183] Park S. G. Lee D. Seo H.-R. Lee S.-A. Kwon J. (2020). Cytotoxic activity of bromodomain inhibitor NVS-CECR2-1 on human cancer cells. Sci. Rep. 10 (1), 16330. 10.1038/s41598-020-73500-7 33004947 PMC7529788

[B184] Patil A. Strom A. R. Paulo J. A. Collings C. K. Ruff K. M. Shinn M. K. (2023). A disordered region controls cBAF activity *via* condensation and partner recruitment. Cell 186 (22), 4936–4955.e26. 10.1016/j.cell.2023.08.032 37788668 PMC10792396

[B185] Paul S. Kuo A. Schalch T. Vogel H. Joshua-Tor L. McCombie W. R. (2013). Chd5 requires PHD-mediated histone 3 binding for tumor suppression. Cell Rep. 3 (1), 92–102. 10.1016/j.celrep.2012.12.009 23318260 PMC3575599

[B186] Pedersen A. K. Pfeiffer A. Karemore G. Akimov V. Bekker-Jensen D. B. Blagoev B. (2021). Proteomic investigation of Cbl and Cbl-b in neuroblastoma cell differentiation highlights roles for SHP-2 and CDK16. iScience 24 (4), 102321. 10.1016/j.isci.2021.102321 33889818 PMC8050387

[B187] Pérez-Pena J. Páez R. Nieto-Jiménez C. Sánchez V. C. Galan-Moya E. M. Pandiella A. (2019). Mapping bromodomains in breast cancer and association with clinical outcome. Sci. Rep. 9 (1), 5734. 10.1038/s41598-019-41934-3 30952871 PMC6450889

[B188] Pérez-Salvia M. Esteller M. (2017). Bromodomain inhibitors and cancer therapy: from structures to applications. Epigenetics 12 (5), 323–339. 10.1080/15592294.2016.1265710 27911230 PMC5453193

[B189] Piovesan D. Necci M. Escobedo N. Monzon A. M. Hatos A. Mičetić I. (2021). MobiDB: intrinsically disordered proteins in 2021. Nucleic acids Res. 49 (D1), D361–D367. 10.1093/nar/gkaa1058 33237329 PMC7779018

[B190] Pongor L. Kormos M. Hatzis C. Pusztai L. Szabó A. Győrffy B. (2015). A genome-wide approach to link genotype to clinical outcome by utilizing next generation sequencing and gene chip data of 6,697 breast cancer patients. Genome Med. 7 (1), 104. 10.1186/s13073-015-0228-1 26474971 PMC4609150

[B191] Povlsen L. K. Beli P. Wagner S. A. Poulsen S. L. Sylvestersen K. B. Poulsen J. W. (2012). Systems-wide analysis of ubiquitylation dynamics reveals a key role for PAF15 ubiquitylation in DNA-damage bypass. Nat. Cell Biol. 14 (10), 1089–1098. 10.1038/ncb2579 23000965

[B192] Pradhan S. K. Su T. Yen L. Jacquet K. Huang C. Côté J. (2016). EP400 deposits H3.3 into promoters and enhancers during gene activation. Mol. Cell 61 (1), 27–38. 10.1016/j.molcel.2015.10.039 26669263 PMC4707986

[B193] Priestley P. Baber J. Lolkema M. P. Steeghs N. de Bruijn E. Shale C. (2019). Pan-cancer whole-genome analyses of metastatic solid tumours. Nature 575 (7781), 210–216. 10.1038/s41586-019-1689-y 31645765 PMC6872491

[B194] Puri P. L. Mercola M. (2012). BAF60 A, B, and Cs of muscle determination and renewal. Genes Dev. 26 (24), 2673–2683. 10.1101/gad.207415.112 23222103 PMC3533072

[B195] Quan J. Yusufzai T. (2014). The tumor suppressor chromodomain helicase DNA-binding protein 5 (CHD5) remodels nucleosomes by unwrapping. J. Biol. Chem. 289 (30), 20717–20726. 10.1074/jbc.M114.568568 24923445 PMC4110282

[B196] Quan J. Adelmant G. Marto J. A. Look A. T. Yusufzai T. (2014). The chromatin remodeling factor CHD5 is a transcriptional repressor of WEE1. PloS One 9 (9), e108066. 10.1371/journal.pone.0108066 25247294 PMC4172601

[B197] Racki L. R. Naber N. Pate E. Leonard J. D. Cooke R. Narlikar G. J. (2014). The histone H4 tail regulates the conformation of the ATP-binding pocket in the SNF2h chromatin remodeling enzyme. J. Mol. Biol. 426 (10), 2034–2044. 10.1016/j.jmb.2014.02.021 24607692 PMC4059342

[B198] Rago F. Elliott G. Li A. Sprouffske K. Kerr G. Desplat A. (2020). The discovery of SWI/SNF chromatin remodeling activity as a novel and targetable dependency in uveal melanoma. Mol. Cancer Ther. 19 (10), 2186–2195. 10.1158/1535-7163.MCT-19-1013 32747420

[B199] Rakesh R. Chanana U. B. Hussain S. Sharma S. Goel K. Bisht D. (2021). Altering mammalian transcription networking with ADAADi: an inhibitor of ATP-dependent chromatin remodeling. Plos One 16 (5), e0251354. 10.1371/journal.pone.0251354 33999958 PMC8128233

[B200] Razavi P. Chang M. T. Xu G. Bandlamudi C. Ross D. S. Vasan N. (2018). The genomic landscape of endocrine-resistant advanced breast cancers. Cancer Cell 34 (3), 427–438.e6. 10.1016/j.ccell.2018.08.008 30205045 PMC6327853

[B201] Remillard D. Buckley D. L. Paulk J. Brien G. L. Sonnett M. Seo H. S. (2017). Degradation of the BAF complex factor BRD9 by heterobifunctional ligands. Angew. Chem. Int. Ed. 56 (21), 5738–5743. 10.1002/anie.201611281 28418626 PMC5967236

[B202] Ronan J. L. Wu W. Crabtree G. R. (2013). From neural development to cognition: unexpected roles for chromatin. Nat. Rev. Genet. 14 (5), 347–359. 10.1038/nrg3413 23568486 PMC4010428

[B203] Rudin C. M. Poirier J. T. Byers L. A. Dive C. Dowlati A. George J. (2019). Molecular subtypes of small cell lung cancer: a synthesis of human and mouse model data. Nat. Rev. Cancer 19 (5), 289–297. 10.1038/s41568-019-0133-9 30926931 PMC6538259

[B204] Ruhl D. D. Jin J. Cai Y. Swanson S. Florens L. Washburn M. P. (2006). Purification of a human SRCAP complex that remodels chromatin by incorporating the histone variant H2A.Z into nucleosomes. Biochemistry 45 (17), 5671–5677. 10.1021/bi060043d 16634648

[B205] Runge J. S. Raab J. R. Magnuson T. (2018). Identification of two distinct classes of the human INO80 complex genome-wide. G3 Genes, Genomes, Genet. 8 (4), 1095–1102. 10.1534/g3.117.300504 29432129 PMC5873900

[B206] Russo J. Russo I. H. (2012). Molecular basis of pregnancy-induced breast cancer prevention. Hormone Mol. Biol. Clin. Investigation 9 (1), 3–10. 10.1515/hmbci-2011-0136 25961350

[B207] Ryan D. P. Sundaramoorthy R. Martin D. Singh V. Owen-Hughes T. (2011). The DNA-binding domain of the Chd1 chromatin-remodelling enzyme contains SANT and SLIDE domains. Embo J. 30 (13), 2596–2609. 10.1038/emboj.2011.166 21623345 PMC3155300

[B208] Sabari B. R. Dall’Agnese A. Boija A. Klein I. A. Coffey E. L. Shrinivas K. (2018). Coactivator condensation at super-enhancers links phase separation and gene control. Science 361 (6400), eaar3958. 10.1126/science.aar3958 29930091 PMC6092193

[B209] Sammak S. Zinzalla G. (2015). Targeting protein–protein interactions (PPIs) of transcription factors: challenges of intrinsically disordered proteins (IDPs) and regions (IDRs). Prog. biophysics Mol. Biol. 119 (1), 41–46. 10.1016/j.pbiomolbio.2015.06.004 26126425

[B210] Samuelson A. V. Narita M. Chan H.-M. Jin J. de Stanchina E. McCurrach M. E. (2005). p400 is required for E1A to promote apoptosis. J. Biol. Chem. 280 (23), 21915–21923. 10.1074/jbc.M414564200 15741165

[B211] Santisteban M. S. Kalashnikova T. Smith M. M. (2000). Histone H2A.Z regulats transcription and is partially redundant with nucleosome remodeling complexes. Cell 103 (3), 411–422. 10.1016/s0092-8674(00)00133-1 11081628

[B212] Schick S. Rendeiro A. F. Runggatscher K. Ringler A. Boidol B. Hinkel M. (2019). Systematic characterization of BAF mutations provides insights into intracomplex synthetic lethalities in human cancers. Nat. Genet. 51 (9), 1399–1410. 10.1038/s41588-019-0477-9 31427792 PMC6952272

[B213] Schneider M. Radoux C. J. Hercules A. Ochoa D. Dunham I. Zalmas L.-P. (2021). The PROTACtable genome. Nat. Rev. Drug Discov. 20 (10), 789–797. 10.1038/s41573-021-00245-x 34285415

[B214] Schubert H. L. Wittmeyer J. Kasten M. M. Hinata K. Rawling D. C. Héroux A. (2013). Structure of an actin-related subcomplex of the SWI/SNF chromatin remodeler. Proc. Natl. Acad. Sci. U. S. A. 110(9)**,** 3345–3350. 10.1073/pnas.1215379110 23401505 PMC3587198

[B215] Schwartz C. J. Pareja F. da Silva E. M. Selenica P. Ross D. S. Weigelt B. (2021). Histologic and genomic features of breast cancers with alterations affecting the SWI/SNF (SMARC) genes. Mod. Pathol. 34 (10), 1850–1859. 10.1038/s41379-021-00837-3 34079072 PMC8448940

[B216] Segala G. Bennesch M. A. Pandey D. P. Hulo N. Picard D. (2016). Monoubiquitination of histone H2B blocks eviction of histone variant H2A. Z from inducible enhancers. Mol. Cell 64 (2), 334–346. 10.1016/j.molcel.2016.08.034 27692985

[B217] Sehdev A. S. Kurman R. J. Kuhn E. Shih Ie M. (2010). Serous tubal intraepithelial carcinoma upregulates markers associated with high-grade serous carcinomas including Rsf-1 (HBXAP), cyclin E and fatty acid synthase. Mod. Pathol. 23 (6), 844–855. 10.1038/modpathol.2010.60 20228782 PMC2879438

[B218] Sen M. Wang X. Hamdan F. H. Rapp J. Eggert J. Kosinsky R. L. (2019). ARID1A facilitates KRAS signaling-regulated enhancer activity in an AP1-dependent manner in colorectal cancer cells. Clin. Epigenetics 11 (1), 92. 10.1186/s13148-019-0690-5 31217031 PMC6585056

[B219] Shah S. P. Roth A. Goya R. Oloumi A. Ha G. Zhao Y. (2012). The clonal and mutational evolution spectrum of primary triple-negative breast cancers. Nature 486 (7403), 395–399. 10.1038/nature10933 22495314 PMC3863681

[B220] Shao F. Guo T. Chua P. J. Tang L. Thike A. A. Tan P. H. (2015). Clinicopathological significance of ARID1B in breast invasive ductal carcinoma. Histopathology 67 (5), 709–718. 10.1111/his.12701 25817822

[B221] Shen X. Mizuguchi G. Hamiche A. Wu C. (2000). A chromatin remodelling complex involved in transcription and DNA processing. Nature 406 (6795), 541–544. 10.1038/35020123 10952318

[B222] Shen X. Xiao H. Ranallo R. Wu W.-H. Wu C. (2003). Modulation of ATP-dependent chromatin-remodeling complexes by inositol polyphosphates. Science 299 (5603), 112–114. 10.1126/science.1078068 12434013

[B223] Shen J. Peng Y. Wei L. Zhang W. Yang L. Lan L. (2015). ARID1A deficiency impairs the DNA damage checkpoint and sensitizes cells to PARP inhibitors. Cancer Discov. 5 (7), 752–767. 10.1158/2159-8290.CD-14-0849 26069190 PMC4497871

[B224] Sheu J. J. Guan B. Choi J. H. Lin A. Lee C. H. Hsiao Y. T. (2010). Rsf-1, a chromatin remodeling protein, induces DNA damage and promotes genomic instability. J. Biol. Chem. 285 (49), 38260–38269. 10.1074/jbc.M110.138735 20923775 PMC2992260

[B225] Sheu J. J. Choi J. H. Guan B. Tsai F. J. Hua C. H. Lai M. T. (2013). Rsf-1, a chromatin remodelling protein, interacts with cyclin E1 and promotes tumour development. J. Pathol. 229 (4), 559–568. 10.1002/path.4147 23378270 PMC4353404

[B226] Siegel R. L. Miller K. D. Wagle N. S. Jemal A. (2023). Cancer statistics, 2023. CA A Cancer J. Clin. 73 (1), 17–48. 10.3322/caac.21763 36633525

[B227] Singh M. Popowicz G. M. Krajewski M. Holak T. A. (2007). Structural ramification for acetyl‐lysine recognition by the bromodomain of human BRG1 protein, a central ATPase of the SWI/SNF remodeling complex. Chembiochem 8 (11), 1308–1316. 10.1002/cbic.200600562 17582821

[B228] Smith R. Sellou H. Chapuis C. Huet S. Timinszky G. (2018). CHD3 and CHD4 recruitment and chromatin remodeling activity at DNA breaks is promoted by early poly(ADP-ribose)-dependent chromatin relaxation. Nucleic Acids Res. 46 (12), 6087–6098. 10.1093/nar/gky334 29733391 PMC6158744

[B229] Sobczak M. Pitt A. R. Spickett C. M. Robaszkiewicz A. (2019). PARP1 Co-regulates EP300–BRG1-dependent transcription of genes involved in breast cancer cell proliferation and DNA repair. Cancers (Basel) 11 (10), 1539. 10.3390/cancers11101539 31614656 PMC6826995

[B230] Sobczak M. Pietrzak J. Płoszaj T. Robaszkiewicz A. (2020). BRG1 activates proliferation and transcription of cell cycle-dependent genes in breast cancer cells. Cancers 12 (2), 349. 10.3390/cancers12020349 32033115 PMC7072512

[B231] Stark C. Breitkreutz B. J. Reguly T. Boucher L. Breitkreutz A. Tyers M. (2006). BioGRID: a general repository for interaction datasets. Nucleic Acids Res. 34 (Database issue), D535–D539. 10.1093/nar/gkj109 16381927 PMC1347471

[B232] Stephens P. J. Tarpey P. S. Davies H. Van Loo P. Greenman C. Wedge D. C. (2012). The landscape of cancer genes and mutational processes in breast cancer. Nature 486 (7403), 400–404. 10.1038/nature11017 22722201 PMC3428862

[B233] Sun Y. Han J. Wang Z. Li X. Sun Y. Hu Z. (2021). Safety and efficacy of Bromodomain and extra-terminal inhibitors for the treatment of hematological malignancies and solid tumors: a systematic study of clinical trials. Front. Pharmacol. 11, 621093. 10.3389/fphar.2020.621093 33574760 PMC7870522

[B234] Sung H. Ferlay J. Siegel R. L. Laversanne M. Soerjomataram I. Jemal A. (2021). Global cancer statistics 2020: GLOBOCAN estimates of incidence and mortality worldwide for 36 cancers in 185 countries. CA Cancer J. Clin. 71 (3), 209–249. 10.3322/caac.21660 33538338

[B235] Svotelis A. Gévry N. Grondin G. Gaudreau L. (2010). H2A. Z overexpression promotes cellular proliferation of breast cancer cells. Cell Cycle 9 (2), 364–370. 10.4161/cc.9.2.10465 20023423

[B236] Szerlong H. Hinata K. Viswanathan R. Erdjument-Bromage H. Tempst P. Cairns B. R. (2008). The HSA domain binds nuclear actin-related proteins to regulate chromatin-remodeling ATPases. Nat. Struct. Mol. Biol. 15 (5), 469–476. 10.1038/nsmb.1403 18408732 PMC2810487

[B237] Takada K. Zhu D. Bird G. H. Sukhdeo K. Zhao J. J. Mani M. (2012). Targeted disruption of the BCL9/β-catenin complex inhibits oncogenic wnt signaling. Sci. Transl. Med. 4 (148), 148ra117. 10.1126/scitranslmed.3003808 22914623 PMC3631420

[B238] Tan S. Ding K. Li R. Zhang W. Li G. Kong X. (2014). Identification of miR-26 as a key mediator of estrogen stimulated cell proliferation by targeting CHD1, GREB1 and KPNA2. Breast cancer Res. 16, R40–13. 10.1186/bcr3644 24735615 PMC4053242

[B239] Tanida I. Ueno T. Kominami E. (2008). LC3 and autophagy. Methods Mol. Biol. 445, 77–88. 10.1007/978-1-59745-157-4_4 18425443

[B240] Taubert S. Gorrini C. Frank S. R. Parisi T. Fuchs M. Chan H.-M. (2004). E2F-dependent histone acetylation and recruitment of the Tip60 acetyltransferase complex to chromatin in late G1. Mol. Cell. Biol. 24 (10), 4546–4556. 10.1128/MCB.24.10.4546-4556.2004 15121871 PMC400446

[B241] Thang N. X. Han D. W. Park C. Lee H. La H. Yoo S. (2023). INO80 function is required for mouse mammary gland development, but mutation alone may be insufficient for breast cancer. Front. Cell Dev. Biol. 11, 1253274. 10.3389/fcell.2023.1253274 38020889 PMC10646318

[B242] Torigoe S. E. Urwin D. L. Ishii H. Smith D. E. Kadonaga J. T. (2011). Identification of a rapidly formed nonnucleosomal histone-DNA intermediate that is converted into chromatin by ACF. Mol. Cell 43 (4), 638–648. 10.1016/j.molcel.2011.07.017 21855802 PMC3160715

[B243] Tozlu S. Girault I. Vacher S. Vendrell J. Andrieu C. Spyratos F. (2006). Identification of novel genes that co-cluster with estrogen receptor alpha in breast tumor biopsy specimens, using a large-scale real-time reverse transcription-PCR approach. Endocrine-related cancer 13 (4), 1109–1120. 10.1677/erc.1.01120 17158757

[B244] Tran H. G. Steger D. J. Iyer V. R. Johnson A. D. (2000). The chromo domain protein chd1p from budding yeast is an ATP-dependent chromatin-modifying factor. Embo J. 19 (10), 2323–2331. 10.1093/emboj/19.10.2323 10811623 PMC384354

[B245] Trojer P. (2022). Targeting BET bromodomains in cancer. Annu. Rev. Cancer Biol. 6, 313–336. 10.1146/annurev-cancerbio-070120-103531

[B246] Tropée R. de la Peña Avalos B. Gough M. Snell C. Duijf P. H. G. Dray E. (2021). The SWI/SNF subunit SMARCD3 regulates cell cycle progression and predicts survival outcome in ER+ breast cancer. Breast Cancer Res. Treat. 185 (3), 601–614. 10.1007/s10549-020-05997-5 33180234 PMC8262116

[B247] van Attikum H. Fritsch O. Hohn B. Gasser S. M. (2004). Recruitment of the INO80 complex by H2A phosphorylation links ATP-dependent chromatin remodeling with DNA double-strand break repair. Cell 119 (6), 777–788. 10.1016/j.cell.2004.11.033 15607975

[B248] VanderLinden R. T. Hemmis C. W. Schmitt B. Ndoja A. Whitby F. G. Robinson H. (2015). Structural basis for the activation and inhibition of the UCH37 deubiquitylase. Mol. Cell 57 (5), 901–911. 10.1016/j.molcel.2015.01.016 25702872 PMC4355076

[B249] Varela I. Tarpey P. Raine K. Huang D. Ong C. K. Stephens P. (2011). Exome sequencing identifies frequent mutation of the SWI/SNF complex gene PBRM1 in renal carcinoma. Nature 469 (7331), 539–542. 10.1038/nature09639 21248752 PMC3030920

[B250] Varga-Weisz P. D. Wilm M. Bonte E. Dumas K. Mann M. Becker P. B. (1997). Chromatin-remodelling factor CHRAC contains the ATPases ISWI and topoisomerase II. Nature 388 (6642), 598–602. 10.1038/41587 9252192

[B251] Vaswani R. G. Huang D. S. Anthony N. Xu L. Centore R. Schiller S. (2025). Discovery of FHD-286, a First-in-Class, orally bioavailable, allosteric dual inhibitor of the brahma homologue (BRM) and brahma-related gene 1 (BRG1) ATPase activity for the treatment of SWItch/Sucrose non-fermentable (SWI/SNF) dependent cancers. J. Med. Chem. 68 (2), 1772–1792. 10.1021/acs.jmedchem.4c02535 39801091

[B252] Wagner S. A. Beli P. Weinert B. T. Nielsen M. L. Cox J. Mann M. (2011). A proteome-wide, quantitative survey of *in vivo* ubiquitylation sites reveals widespread regulatory roles. Mol. Cell Proteomics 10 (10), M111.013284. 10.1074/mcp.M111.013284 21890473 PMC3205876

[B253] Wang J. Chen H. Fu S. Xu Z.-M. Sun K.-L. Fu W.-N. (2011). The involvement of CHD5 hypermethylation in laryngeal squamous cell carcinoma. Oral Oncol. 47 (7), 601–608. 10.1016/j.oraloncology.2011.05.003 21636313

[B254] Wang S. Xia P. Ye B. Huang G. Liu J. Fan Z. (2013). Transient activation of autophagy *via* Sox2-mediated suppression of mTOR is an important early step in reprogramming to pluripotency. Cell stem Cell 13 (5), 617–625. 10.1016/j.stem.2013.10.005 24209762

[B255] Wang L. Du Y. Ward J. M. Shimbo T. Lackford B. Zheng X. (2014). INO80 facilitates pluripotency gene activation in embryonic stem cell self-renewal, reprogramming, and blastocyst development. Cell Stem Cell 14 (5), 575–591. 10.1016/j.stem.2014.02.013 24792115 PMC4154226

[B256] Wang X. Wang S. Troisi E. C. Howard T. P. Haswell J. R. Wolf B. K. (2019). BRD9 defines a SWI/SNF sub-complex and constitutes a specific vulnerability in malignant rhabdoid tumors. Nat. Commun. 10 (1), 1881. 10.1038/s41467-019-09891-7 31015438 PMC6479050

[B257] Wang J. He C. Gao P. Wang S. Lv R. Zhou H. (2020a). HNF1B-mediated repression of SLUG is suppressed by EZH2 in aggressive prostate cancer. Oncogene 39 (6), 1335–1346. 10.1038/s41388-019-1065-2 31636385 PMC7002300

[B258] Wang Y. Chen Y. Bao L. Zhang B. Wang J. E. Kumar A. (2020b). CHD4 promotes breast cancer progression as a coactivator of hypoxia-inducible factors. Cancer Res. 80 (18), 3880–3891. 10.1158/0008-5472.CAN-20-1049 32699137 PMC7501193

[B259] Wanior M. Krämer A. Knapp S. Joerger A. C. (2021). Exploiting vulnerabilities of SWI/SNF chromatin remodelling complexes for cancer therapy. Oncogene 40 (21), 3637–3654. 10.1038/s41388-021-01781-x 33941852 PMC8154588

[B260] Wei F.-Z. Cao Z. Wang X. Wang H. Cai M.-Y. Li T. (2015). Epigenetic regulation of autophagy by the methyltransferase EZH2 through an MTOR-dependent pathway. Autophagy 11 (12), 2309–2322. 10.1080/15548627.2015.1117734 26735435 PMC4835210

[B261] Wei M.-T. Chang Y.-C. Shimobayashi S. F. Shin Y. Strom A. R. Brangwynne C. P. (2020). Nucleated transcriptional condensates amplify gene expression. Nat. Cell Biol. 22 (10), 1187–1196. 10.1038/s41556-020-00578-6 32929202

[B262] Weinstein J. N. Collisson E. A. Mills G. B. Shaw K. R. Ozenberger B. A. Ellrott K. (2013). The cancer genome atlas pan-cancer analysis project. Nat. Genet. 45 (10), 1113–1120. 10.1038/ng.2764 24071849 PMC3919969

[B263] Wicks S. J. Haros K. Maillard M. Song L. Cohen R. E. Dijke P. T. (2005). The deubiquitinating enzyme UCH37 interacts with Smads and regulates TGF-β signalling. Oncogene 24 (54), 8080–8084. 10.1038/sj.onc.1208944 16027725

[B264] Wicks S. Grocott T. Haros K. Maillard M. Dijke P. t. Chantry A. (2006). Reversible ubiquitination regulates the Smad/TGF-β signalling pathway. Portland Press Ltd 34 (pt 5), 761–763. 17052192 10.1042/BST0340761

[B265] Willhoft O. Bythell-Douglas R. McCormack E. A. Wigley D. B. (2016). Synergy and antagonism in regulation of recombinant human INO80 chromatin remodeling complex. Nucleic Acids Res. 44 (17), 8179–8188. 10.1093/nar/gkw509 27257055 PMC5041457

[B266] Williamson C. T. Miller R. Pemberton H. N. Jones S. E. Campbell J. Konde A. (2016). ATR inhibitors as a synthetic lethal therapy for tumours deficient in ARID1A. Nat. Commun. 7 (1), 13837. 10.1038/ncomms13837 27958275 PMC5159945

[B267] Wilson B. G. Roberts C. W. (2011). SWI/SNF nucleosome remodellers and cancer. Nat. Rev. Cancer 11 (7), 481–492. 10.1038/nrc3068 21654818

[B268] Wu J. I. (2012). Diverse functions of ATP-dependent chromatin remodeling complexes in development and cancer. Acta Biochim. Biophys. Sin. 44 (1), 54–69. 10.1093/abbs/gmr099 22194014

[B269] Wu X. Zhu Z. Li W. Fu X. Su D. Fu L. (2012). Chromodomain helicase DNA binding protein 5 plays a tumor suppressor role in human breast cancer. Breast Cancer Res. 14, R73–16. 10.1186/bcr3182 22569290 PMC3446335

[B270] Wu Q. Madany P. Akech J. Dobson J. R. Douthwright S. Browne G. (2015). The SWI/SNF ATPases are required for triple negative breast cancer cell proliferation. J. Cell. Physiol. 230 (11), 2683–2694. 10.1002/jcp.24991 25808524 PMC4516601

[B271] Wu Q. Madany P. Dobson J. R. Schnabl J. M. Sharma S. Smith T. C. (2016a). The BRG1 chromatin remodeling enzyme links cancer cell metabolism and proliferation. Oncotarget 7 (25), 38270–38281. 10.18632/oncotarget.9505 27223259 PMC5122388

[B272] Wu Q. Sharma S. Cui H. LeBlanc S. E. Zhang H. Muthuswami R. (2016b). Targeting the chromatin remodeling enzyme BRG1 increases the efficacy of chemotherapy drugs in breast cancer cells. Oncotarget 7 (19), 27158–27175. 10.18632/oncotarget.8384 27029062 PMC5053639

[B273] Wu Q. Lian J. B. Stein J. L. Stein G. S. Nickerson J. A. Imbalzano A. N. (2017). The BRG1 ATPase of human SWI/SNF chromatin remodeling enzymes as a driver of cancer. Epigenomics 9 (6), 919–931. 10.2217/epi-2017-0034 28521512 PMC5705788

[B274] Wu C. Lyu J. Yang E. J. Liu Y. Zhang B. Shim J. S. (2018). Targeting AURKA-CDC25C axis to induce synthetic lethality in ARID1A-deficient colorectal cancer cells. Nat. Commun. 9 (1), 3212. 10.1038/s41467-018-05694-4 30097580 PMC6086874

[B275] Xia W. Nagase S. Montia A. G. Kalachikov S. M. Keniry M. Su T. (2008). BAF180 is a critical regulator of p21 induction and a tumor suppressor mutated in breast cancer. Cancer Res. 68 (6), 1667–1674. 10.1158/0008-5472.Can-07-5276 18339845 PMC2915562

[B276] Xiao H. Sandaltzopoulos R. Wang H. M. Hamiche A. Ranallo R. Lee K. M. (2001). Dual functions of largest NURF subunit NURF301 in nucleosome sliding and transcription factor interactions. Mol. Cell 8 (3), 531–543. 10.1016/s1097-2765(01)00345-8 11583616

[B277] Xiao L. Parolia A. Qiao Y. Bawa P. Eyunni S. Mannan R. (2022). Targeting SWI/SNF ATPases in enhancer-addicted prostate cancer. Nature 601 (7893), 434–439. 10.1038/s41586-021-04246-z 34937944 PMC8770127

[B278] Xie C.-R. Li Z. Sun H.-G. Wang F.-Q. Sun Y. Zhao W.-X. (2015). Mutual regulation between CHD5 and EZH2 in hepatocellular carcinoma. Oncotarget 6 (38), 40940–40952. 10.18632/oncotarget.5724 26517514 PMC4747380

[B279] Xie F. Zhou X. Ran Y. Li R. Zou J. Wan S. (2025). Targeting FOXM1 condensates reduces breast tumour growth and metastasis. Nature 638 (8052), 1112–1121. 10.1038/s41586-024-08421-w 39814884

[B280] Xu G. Chhangawala S. Cocco E. Razavi P. Cai Y. Otto J. E. (2020). ARID1A determines luminal identity and therapeutic response in estrogen-receptor-positive breast cancer. Nat. Genet. 52 (2), 198–207. 10.1038/s41588-019-0554-0 31932695 PMC7341683

[B281] Xue Y. Wong J. Moreno G. T. Young M. K. Côté J. Wang W. (1998). NURD, a novel complex with both ATP-dependent chromatin-remodeling and histone deacetylase activities. Mol. Cell 2 (6), 851–861. 10.1016/s1097-2765(00)80299-3 9885572

[B282] Xue Y. Canman J. C. Lee C. S. Nie Z. Yang D. Moreno G. T. (2000). The human SWI/SNF-B chromatin-remodeling complex is related to yeast rsc and localizes at kinetochores of mitotic chromosomes. Proc. Natl. Acad. Sci. U. S. A. 97 (24), 13015–13020. 10.1073/pnas.240208597 11078522 PMC27170

[B283] Xue Y. Van C. Pradhan S. K. Su T. Gehrke J. Kuryan B. G. (2015). The Ino80 complex prevents invasion of euchromatin into silent chromatin. Genes Dev. 29 (4), 350–355. 10.1101/gad.256255.114 25691465 PMC4335291

[B284] Xue Y. Meehan B. Fu Z. Wang X. Q. D. Fiset P. O. Rieker R. (2019a). SMARCA4 loss is synthetic lethal with CDK4/6 inhibition in non-small cell lung cancer. Nat. Commun. 10 (1), 557. 10.1038/s41467-019-08380-1 30718506 PMC6362083

[B285] Xue Y. Meehan B. Macdonald E. Venneti S. Wang X. Q. D. Witkowski L. (2019b). CDK4/6 inhibitors target SMARCA4-determined cyclin D1 deficiency in hypercalcemic small cell carcinoma of the ovary. Nat. Commun. 10 (1), 558. 10.1038/s41467-018-06958-9 30718512 PMC6361890

[B286] Yadon A. N. Tsukiyama T. (2011). SnapShot: chromatin remodeling: ISWI. Cell 144 (3), 453–453.e1. 10.1016/j.cell.2011.01.019 21295704

[B287] Yamamoto S. Tsuda H. Takano M. Tamai S. Matsubara O. (2012). PIK3CA mutations and loss of ARID1A protein expression are early events in the development of cystic ovarian clear cell adenocarcinoma. Virchows Arch. 460 (1), 77–87. 10.1007/s00428-011-1169-8 22120431

[B288] Yamashita N. Morimoto Y. Fushimi A. Ahmad R. Bhattacharya A. Daimon T. (2023). MUC1-C dictates PBRM1-Mediated chronic induction of interferon signaling, DNA damage resistance, and immunosuppression in triple-negative breast cancer. Mol. Cancer Res. 21 (3), 274–289. 10.1158/1541-7786.Mcr-22-0772 36445328 PMC9975675

[B289] Yang D. Denny S. K. Greenside P. G. Chaikovsky A. C. Brady J. J. Ouadah Y. (2018). Intertumoral heterogeneity in SCLC is influenced by the cell type of origin. Cancer Discov. 8 (10), 1316–1331. 10.1158/2159-8290.Cd-17-0987 30228179 PMC6195211

[B290] Yang Y. Liu L. Fang M. Bai H. Xu Y. (2019). The chromatin remodeling protein BRM regulates the transcription of tight junction proteins: implication in breast cancer metastasis. Biochimica Biophysica Acta (BBA) - Gene Regul. Mech. 1862 (5), 547–556. 10.1016/j.bbagrm.2019.03.002 30946989

[B291] Yates L. R. Knappskog S. Wedge D. Farmery J. H. R. Gonzalez S. Martincorena I. (2017). Genomic evolution of breast cancer metastasis and relapse. Cancer Cell 32 (2), 169–184.e7. 10.1016/j.ccell.2017.07.005 28810143 PMC5559645

[B292] Ye Y. Xiao Y. Wang W. Wang Q. Yearsley K. Wani A. A. (2009). Inhibition of expression of the chromatin remodeling gene, SNF2L, selectively leads to DNA damage, growth inhibition, and cancer cell death. Mol. Cancer Res. 7 (12), 1984–1999. 10.1158/1541-7786.Mcr-09-0119 19996304

[B293] Ye F. Huang J. Wang H. Luo C. Zhao K. (2019). Targeting epigenetic machinery: emerging novel allosteric inhibitors. Pharmacol. Ther. 204, 107406. 10.1016/j.pharmthera.2019.107406 31521697

[B294] Yu B. Huang Z. Zhang M. Dillard D. R. Ji H. (2013). Rational design of small-molecule inhibitors for β-catenin/T-cell factor protein-protein interactions by bioisostere replacement. ACS Chem. Biol. 8 (3), 524–529. 10.1021/cb300564v 23272635

[B295] Yu B. Swatkoski S. Holly A. Lee L. C. Giroux V. Lee C.-S. (2015). Oncogenesis driven by the Ras/Raf pathway requires the SUMO E2 ligase Ubc9. Proc. Natl. Acad. Sci. U. S. A. 112 (14), E1724–E1733. 10.1073/pnas.1415569112 25805818 PMC4394293

[B296] Zang Z. J. Cutcutache I. Poon S. L. Zhang S. L. McPherson J. R. Tao J. (2012). Exome sequencing of gastric adenocarcinoma identifies recurrent somatic mutations in cell adhesion and chromatin remodeling genes. Nat. Genet. 44 (5), 570–574. 10.1038/ng.2246 22484628

[B297] Zhang F.-L. Li D.-Q. (2022). Targeting chromatin-remodeling factors in cancer cells: promising molecules in cancer therapy. Int. J. Mol. Sci. 23 (21), 12815. 10.3390/ijms232112815 36361605 PMC9655648

[B298] Zhang X. Zhang Y. Yang Y. Niu M. Sun S. Ji H. (2012). Frequent low expression of chromatin remodeling gene ARID1A in breast cancer and its clinical significance. Cancer Epidemiol. 36 (3), 288–293. 10.1016/j.canep.2011.07.006 21889920

[B299] Zhang J. Hou S. You Z. Li G. Xu S. Li X. (2021). Expression and prognostic values of ARID family members in breast cancer. Aging (Albany NY) 13 (4), 5621–5637. 10.18632/aging.202489 33592583 PMC7950271

[B300] Zhang B. Liu Q. Wen W. Gao H. Wei W. Tang A. (2022a). The chromatin remodeler CHD6 promotes colorectal cancer development by regulating TMEM65-mediated mitochondrial dynamics *via* EGF and Wnt signaling. Cell Discov. 8 (1), 130. 10.1038/s41421-022-00478-z 36473865 PMC9727023

[B301] Zhang M. Liu Z. Z. Aoshima K. Cai W. L. Sun H. Xu T. (2022b). CECR2 drives breast cancer metastasis by promoting NF-κB signaling and macrophage-mediated immune suppression. Sci. Transl. Med. 14 (630), eabf5473. 10.1126/scitranslmed.abf5473 35108062 PMC9003667

[B302] Zhao D. Lu X. Wang G. Lan Z. Liao W. Li J. (2017). Synthetic essentiality of chromatin remodelling factor CHD1 in PTEN-deficient cancer. Nature 542 (7642), 484–488. 10.1038/nature21357 28166537 PMC5448706

[B303] Zhou Q. Huang J. Zhang C. Zhao F. Kim W. Tu X. (2020). The bromodomain containing protein BRD-9 orchestrates RAD51–RAD54 complex formation and regulates homologous recombination-mediated repair. Nat. Commun. 11 (1), 2639. 10.1038/s41467-020-16443-x 32457312 PMC7251110

[B304] Zhu P. Wang Y. He L. Huang G. Du Y. Zhang G. (2015). ZIC2-dependent OCT4 activation drives self-renewal of human liver cancer stem cells. J. Clin. Investigation 125 (10), 3795–3808. 10.1172/JCI81979 26426078 PMC4607118

[B305] Zhu Y. Yan C. Wang X. Xu Z. Lv J. Xu X. (2022). Pan-cancer analysis of ARID family members as novel biomarkers for immune checkpoint inhibitor therapy. Cancer Biol. Ther. 23 (1), 104–111. 10.1080/15384047.2021.2011643 35239432 PMC8896200

[B306] Zinzalla G. (2016). A new way forward in cancer drug discovery: inhibiting the SWI/SNF chromatin remodelling complex. ChemBioChem 17 (8), 677–682. 10.1002/cbic.201500565 26684344

[B307] Zoppi V. Hughes S. J. Maniaci C. Testa A. Gmaschitz T. Wieshofer C. (2018). Iterative design and optimization of initially inactive proteolysis targeting chimeras (PROTACs) identify VZ185 as a potent, fast, and selective von Hippel–Lindau (VHL) based dual degrader probe of BRD9 and BRD7. J. Med. Chem. 62 (2), 699–726. 10.1021/acs.jmedchem.8b01413 30540463 PMC6348446

